# Resurgence of large-scale influenza A (H1N1) outbreaks: Modeling the interplay of transmission, loss of immunity, and vaccination

**DOI:** 10.1016/j.idm.2026.08.001

**Published:** 2026-08-26

**Authors:** Chanaka Kottegoda, Claudia T. Codeço, Claudio J. Struchiner, Lucas M. Stolerman

**Affiliations:** a Department of Mathematics and Physics, Marshall University, Huntington, WV, USA; b Programa de Computação Científica, Fundação Oswaldo Cruz, Rio de Janeiro, Brazil; c School of Applied Mathematics (EMAp FGV), Brazil; d Department of Mathematics, Oklahoma State University, Stillwater, OK, USA

**Keywords:** A(H1N1) influenza, Resurgent outbreaks, Mechanistic modeling

## Abstract

Influenza is a highly transmissible respiratory virus that can cause devastating pandemics. The 2009 influenza pandemic, caused by a strain of A(H1N1) virus, resulted in millions of cases and hundreds of thousands of deaths worldwide. In the following years, many regions experienced waves driven by environmental conditions, waning immunity, and other factors. More dramatically, several locations saw unpredicted large resurgent outbreaks, yet the mechanisms behind these severe resurgences remain poorly understood. Despite an extensive literature of epidemic models aimed at explaining and predicting H1N1 dynamics, mathematical frameworks specifically designed to address why these large outbreaks recur years after an initial epidemic are still lacking. Here we address this gap by proposing a mechanistic model with time-varying rates of infection, loss of immunity, and vaccination. In particular, we explore two mathematical functions to model dynamic loss of immunity, capturing how viral evolution may reduce the duration of immune protection. Numerical simulations show that the model reproduces key features of H1N1 case data, and that delayed resurgence is associated with longer immunity periods and higher vaccine efficacy. We also fit the model to data from Brazil, Turkey, Iran, New Zealand, the United States, Colombia, South Africa, and Croatia, demonstrating its ability to capture resurgence patterns. Fitted infection rates exhibited a wide range of temporal profiles, from regular annual cycles to higher-frequency oscillations and gradual changes, suggesting that transmission is driven by factors beyond seasonal influences. The model also inferred dynamic loss-of-immunity rates, representing different effects of antigenic drift. To our knowledge, this is the first mechanistic modeling study specifically aimed at investigating large resurgent H1N1 epidemics across multiple locations. Our findings highlight the need for context-specific models and surveillance systems that account for the complex interplay among environmental drivers, population immunity, and viral mutation driving H1N1 dynamics.

## 1 Introduction

Influenza pandemics have caused large economic and health impacts throughout history and remain an important threat. The last pandemic virus, the influenza A(H1N1), emerged in the spring of 2009 in central Mexico and rapidly spread worldwide (Harrington et al., 2021). The Centers for Disease Control and Prevention (CDC) reported the first US cases in April 2009, and the World Health Organization (WHO) declared a pandemic on June 11, 2009 (Centers for Disease Control and Prevention). From April 2009 to April 2010, the CDC reported 60.8 million cases, 274,304 hospitalizations, and 12,469 deaths in the United States alone (National Library of Medicine, 2020). Globally, many countries – including Brazil, China, Australia, and the United Kingdom – experienced significant surges between 2009 and 2010 (Kleber de Oliveira et al., 2009; Liang et al., 2012; Pebody et al., 2010; Torres Codeço et al., 2012; Weeramanthri et al., 2010). The 2009 H1N1 virus has continued to circulate, causing subsequent outbreaks (Centers for Disease Control and Prevention, 2019).

Since its emergence, large H1N1 outbreaks have occurred worldwide, often reaching incidence levels comparable to those of the initial 2009 pandemic. For instance, an estimated 30 million influenza illnesses, 13 million medical visits, 347,000 hospitalizations, and 38,000 deaths occurred in the United States during the 2013–2014 influenza season, when 2009 H1N1 viruses were the predominant circulating strains, primarily affecting young and middle-aged adults (Centers for Disease Control and Prevention, 2014a; Centers for Disease Control and Prevention, 2014b; Linderman et al., 2014). In 2016, Brazil experienced a severe outbreak with more than a thousand confirmed deaths (The Disease Daily, 2016). Iran similarly faced a substantial resurgence during the 2015–2016 season, with elevated case counts and mortality (Babamahmoodi et al., 2017; Sedghian et al., 2025). Similar spikes were reported in Turkey (2016), South Africa (2011), Croatia (2011), among other regions (Balkan Insight, 2011; European Centre for Disease Prevention and Control, 2011; Guldemir et al., 2019).

The mechanisms underlying sustained H1N1 transmission after the first wave are understood to an extent. During the 2009 outbreaks, susceptible populations were not fully depleted, allowing for subsequent waves of infection. As the number of susceptible individuals declined below critical thresholds, outbreaks became less frequent, resulting in “skip-and-resurgence” patterns (Stone et al., 2007). Complex oscillations in influenza epidemics are thought to arise from a combination of epidemiological, immunological (humoral), and seasonal drivers, many of which have been successfully incorporated into mathematical models.

Seasonal variations in transmissibility are known to drive periodic influenza activity in temperate regions. Dushoff et al. introduced a sinusoidal infection rate with an annual period to demonstrate that large oscillations in incidence can be caused by small seasonal changes in transmission, amplified by dynamical resonance (Dushoff et al., 2004). Among climatic drivers, the role of humidity in modulating transmission has been a major focus of experimental studies (Lowen et al., 2007; Shaman & Kohn, 2009). By incorporating absolute humidity into models of temporally forced infection rates, Shaman et al. demonstrated an association between anomalously low humidity and the onset of increased wintertime influenza-related mortality in the United States (Shaman et al., 2010). More recently, Dalziel et al. utilized an exponential, humidity-dependent transmission formulation to accurately predict observed differences in the intensity of influenza epidemics across diverse urban centers (Dalziel et al., 2018). In tropical and subtropical regions, modeling efforts have incorporated temperature alongside humidity to capture seasonality that departs from the temperate single annual peak (Deyle et al., 2016; Mahmud et al., 2023; Yuan et al., 2021).

The role of viral evolution shaping influenza dynamics has been a topic of intense modeling. Earn et al. (Earn et al., 2002) noted the limitations of standard SIR frameworks in capturing antigenic drift, building on early evolutionary–epidemiological models such as the one proposed by Pease in 1987, which linked the replenishment of susceptibles to the rate of antigenic change in the circulating virus (Pease, 1987). An SIRS model with surges of immunity loss modeled by discrete jumps was proposed by Yaari et al. (Yaari et al., 2013) and further utilized to describe multiannual seasonal influenza outbreaks (Bock Axelsen et al., 2014). More recently, Du et al. (Du et al., 2017) developed an evolution-informed forecasting model integrating viral genomic data to inform changes in population susceptibility from antigenic evolution. A stochastic approach proposed by Woolthuis et al. (Woolthuis et al., 2017), in which the duration of immunity was drawn from a probability distribution in each season, provided evidence that variability in the immunity period can increase the height of epidemic peaks.

Poor vaccination coverage, suboptimal vaccine efficacy, and demographic shifts further contribute to sustaining recurrent influenza outbreaks (Monto, 2010; Skowronski et al., 2016). Addressing demographic heterogeneity from an early intervention perspective, Medlock and Galvani (Jan et al., 2009) integrated survey-based contact data into a transmission model to determine the optimal allocation of vaccines among distinct age groups. Scaling these targeted interventions to a global level during the 2009 pandemic, Bajardi et al. (Bajardi et al., 2009) employed a metapopulation model driven by real-world mobility data to assess the effectiveness of massive vaccination campaigns. Their findings highlighted the critical need for prioritized vaccination strategies, concluding that additional interventions are often required alongside vaccination to successfully delay epidemic peaks. Building on this foundational understanding of population structure, Bansal et al. (Bansal et al., 2010) later utilized network models to demonstrate how these age-specific contact patterns and evolving transmission behaviors shape the burden of infection across successive epidemic waves.

The coupled roles of immunity, infection, and vaccination have been examined across a range of modeling frameworks. Recent work has introduced immuno-epidemiological models that explicitly incorporate immunity and infection ages to characterize epidemic dynamics (Angelov et al., 2024; Raimund et al., 2025). Mechanistic approaches have further investigated how waning immunity can shape viral evolution, demonstrating that immune-escape variants may arise dynamically through feedbacks between population-level immunity and viral adaptation (Bull et al., 2025; Frost Nielsen et al., 2025). Other studies have shown that distinct epidemiological patterns can emerge from differences in the duration of vaccine-acquired versus infection-acquired immunity (Leung et al., 2018), and that models with multiple partial-immunity classes are capable of generating sustained epidemic waves (Lai & Gog, 2024). Extensions incorporating graded susceptibility have yielded explicit conditions on vaccine supply required to maintain disease-free states (El Khalifi & Britton, 2023), while frameworks allowing immunity boosting upon re-exposure highlight the critical role of immunity duration in shaping transmission dynamics (Opoku-Sarkodie et al., 2022). In the specific context of influenza following the 2009 pandemic, a comprehensive global analysis by He et al. identified synchronization patterns and hypothesized that these arise from the combined effects of seasonal forcing, strain-specific loss of immunity, and vaccination coverage (He et al., 2015). Despite these advances, to our knowledge, no mechanistic model has yet been applied to specifically investigate the large resurgent H1N1 epidemics observed across multiple locations.

Here, we address this gap by asking: how do transmission, loss of immunity, and vaccination shape the timing and magnitude of large resurgent H1N1 outbreaks? To investigate this question, we developed a mathematical model that differs from earlier models in how it represents these mechanisms. Rather than fixing transmission to a canonical annual cycle, we construct a flexible sinusoidal rate that may capture non-canonical dynamics beyond seasonal influences. We represent time-varying loss of immunity through a linear increase or a single jump, the latter a simplification of the multi-jump formulation used by Yaari et al. (Yaari et al., 2013). Vaccination is modeled explicitly as a rectangular seasonal pulse rather than as a constant rate. Through numerical simulations, we identify parameter regimes that influence resurgence timing and show that our model reproduces key patterns observed in H1N1 time series data. By fitting the model to data from nine locations, we demonstrate that it captures the timing and key features of large resurgent outbreaks, reveal that transmission dynamics extend beyond simple seasonal forcing, and uncover location-specific patterns in immunity loss that suggest distinct antigenic drift trajectories across regions.

## 2 Materials and methods

### 2.1 Epidemiological and demographic datasets

We identified locations that experienced large resurgent H1N1 outbreaks worldwide by examining open datasets with reported H1N1 case counts. Epidemiological data from HHS Regions 2 and 6 were obtained from the Fluview interactive system (Centers for Disease Control and Prevention, 2023). H1N1 case counts for São Paulo were collected from the Brazilian Information System for Influenza Epidemiological Surveillance (SIVEP). The rest of the epidemiological data for other locations were obtained from FluNet (Flahault et al., 1998), the World Health Organization's (WHO) influenza surveillance system, which provides real-time, publicly accessible epidemiological data. For each location, we also collected population data from 2009 to the year of the large resurgence outbreak, sourced from Worldbank.org (World bank). For datasets and further details, we refer the reader to the supplementary material.

Defining what constitutes a large resurgence is challenging and can be approached in multiple ways. Here, a large resurgent H1N1 outbreak is defined as one whose epidemic peak (maximum number of reported cases) exceeds 50% of the major peak observed during the 2009 pandemic. A comparative approach has been employed to define epidemic “skip” years as seasons in which case numbers dropped by more than an order of magnitude from the previous year (He et al., 2015). However, an outbreak that avoids the ‘skip’ classification is not necessarily a *large* one. At the other extreme, the 2009 H1N1pdm pandemic strain encountered a largely susceptible population globally. Hence, requiring resurgent outbreaks to match 2009 levels would be overly restrictive. Between these bounds, the 50% threshold strikes a balance, identifying epidemiologically significant events achieved in the following years.

**Fig. 1:**
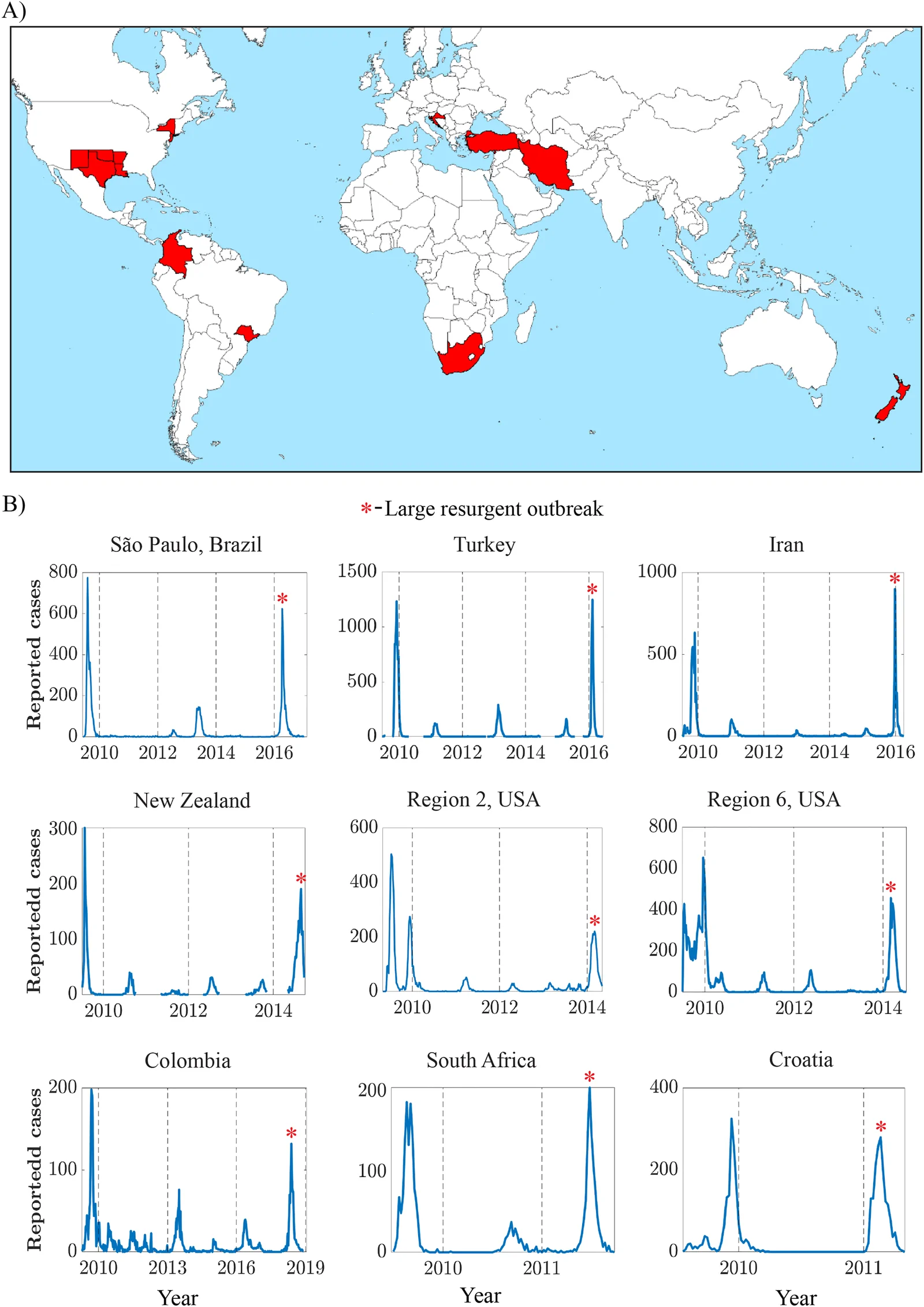
**Weekly reported A(H1N1) influenza cases in selected regions.** (A) Geographical locations of nine selected regions from eight countries that experienced a large resurgent H1N1 outbreak after the 2009 pandemic. (B) Weekly reported H1N1 cases in the selected regions, starting from the 2009 pandemic until the year of the large resurgent outbreak (marked by a red star). The visible gaps between the blue curves in New Zealand and Turkey indicate missing data.

Major outbreaks in 2010 were not included and classified as late-pandemic activity rather than resurgences. Fig. 1 A) displays the nine selected locations/countries: São Paulo State in Brazil, Turkey, Iran, New Zealand, the U.S. Department of Health and Human Services (HHS) Regions 2 and 6, Colombia, South Africa, and Croatia. The HHS Region 2 includes the states of New Jersey and New York, as well as Puerto Rico and the U.S. Virgin Islands. The HHS Region 6 comprises the states of Arkansas, Louisiana, New Mexico, Oklahoma, and Texas.

Fig. 1 B) displays the time series of weekly reported H1N1 cases between 2009 and the first large resurgent outbreaks for each location. Some epidemiological datasets exhibited gaps in the data. In particular, New Zealand had 103 missing data points out of 273 weeks, Iran had eight missing data points out of 351 weeks, and Turkey had 69 missing data points out of 364 weeks. In this work, we did not perform data imputation or estimation for the missing values; only the available data were used in the model-fitting analysis. In Fig. 1 B), the large resurgent outbreaks are marked with a red star for each location.

### 2.2 Mathematical model

**Fig. 2:**
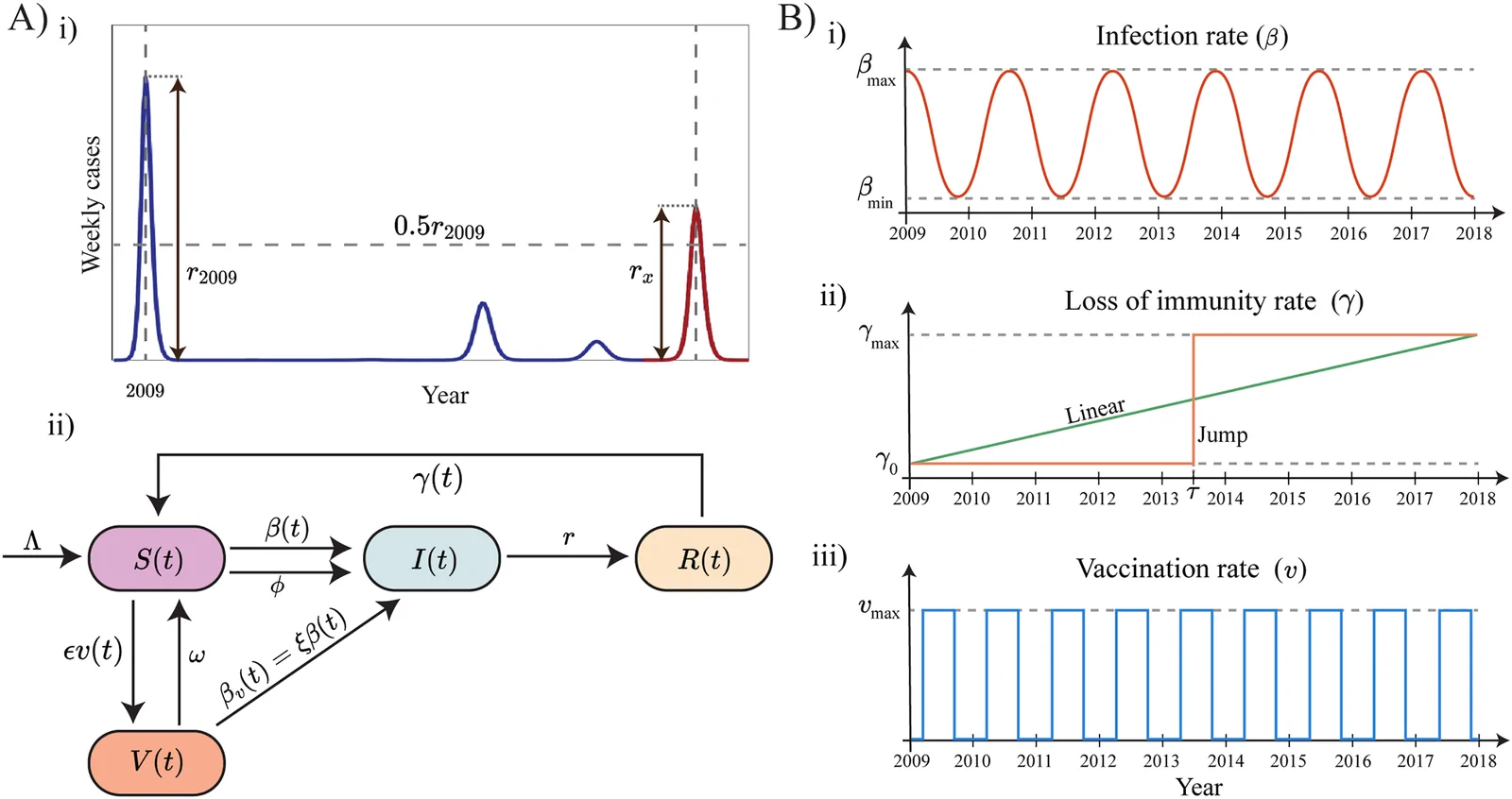
**Large resurgent H1N1 outbreaks and our proposed SVIRS mathematical model.** A) i) The resurgence of large H1N1 influenza outbreak in year *x* is defined by the condition *r*_*x*_ > 0.5*r*_2009_. Here *r*_2009_ represents the epidemic peak during the 2009 H1N1 pandemic, while *r*_*x*_ is the same quantity in year *x*. A) ii) A compartmental diagram of our SVIRS model. A population is divided into susceptible (S), vaccinated (V), infectious (I), and recovered individuals (R). The rates of Infection, loss of immunity, and vaccination are assumed to change over time. B) i) A sine function was used to approximate the periodic infection rate *β*(*t*), with minimum and maximum values *β*_min_ and *β*_max_. B) ii). Linear and Jump functions represent different effects of antigenic drift on the loss-of-immunity rate *γ*(*t*) that increases from *γ*_0_ to *γ*_max_. B) iii). A rectangular pulse function represented the vaccination rate, which increases to *v*_max_ during vaccination periods.

We developed a modification of the classic SIRS model with vaccination (SVIRS), combining time-varying rates of transmission, loss of immunity, and vaccination to explore the dynamics of large resurgent H1N1 outbreaks. The compartmental diagram of our model is illustrated in Fig. 2 A) i). To maintain mathematical simplicity, the model omits age-structured dynamics, heterogeneous contact patterns, and exposure periods. The governing equations are given by the following system:

(1) $$\left(\begin{array}{cc} \dot{S} & =\Lambda -\beta \left(t\right)S\frac{I}{N}-\phi S+\gamma \left(t\right)R-\epsilon v\left(t\right)S+\omega V,\\ \dot{V} & =\epsilon v\left(t\right)S-\omega V-\beta_{v}\left(t\right)V\frac{I}{N},\\ \dot{I} & =\beta \left(t\right)S\frac{I}{N}+\phi S-rI+\beta_{v}\left(t\right)V\frac{I}{N},\\ \dot{R} & =rI-\gamma \left(t\right)R. \end{array}\right)$$

Here, *S*, *V*, *I*, and *R* represent the susceptible, vaccinated, infectious, and recovered individuals, respectively. Overdots denote derivatives with respect to time. The time-varying rates are assumed to reflect dynamic changes driven by complex biological factors, which are discussed in detail in the next subsection. Here, *β*(*t*) denotes the infection rate at which susceptible individuals become infectious. The parameter *γ*(*t*) represents the loss-of-immunity rate, and *v*(*t*) the vaccination rate. Additionally, *β*_*v*_(*t*) = *ξβ*(*t*) denotes the infection rate at which vaccinated individuals become infectious. The factor *ξ* between 0 and 1 represents the vaccine efficacy in terms of the residual susceptibility of vaccinated individuals to infection. Hence, *ξ* = 0 corresponds to a vaccine that confers perfect protection. The recruitment rate Λ is constant and the total population *N*(*t*) satisfies the differential equation $\dot{N}=\Lambda$, leading to *N*(*t*) = Λ*t* + *N*_0_, where *N*_0_ denotes the total initial population. This linear trend provides a suitable approximation for the complex demographic evolution observed in the selected locations during the initial years following the 2009 H1N1 pandemic (see supplementary material). Natural recovery rates are represented by the parameter *r*, while *ω* describes the rate at which vaccine-induced immunity wanes. Finally, *ϵ* and *ϕ* represent the vaccine effectiveness (i.e., the fraction of vaccinated susceptibles who are effectively protected) and a small importation rate, respectively. We list all fixed (time-independent) parameters in Table 1 (see supplementary material for details).

**Table 1:** Time-independent model parameters: description and values.

Parameter	Description	Value
Λ	Recruitment rate into the susceptible class	Location-specific; estimated from demographic data (see supplementary material)
*r*	Natural recovery rate	1.17 week^−1^ (Novel Swine-Origin Influenza A (H1N1) Virus Investigation Team, 2009)
*ϕ*	Importation rate	10^−6^ week^−1^ (one imported case per million susceptibles per week)
*ϵ*	Vaccine effectiveness	0.7 (Osterholm et al., 2012)
*ω*	Waning rate of vaccine-induced immunity	0.019 week^−1^ (Young et al., 2018)

### 2.3 Model assumptions for time-varying rates

*Periodic infection rates.* Influenza transmission depends on multiple environmental and social factors with seasonal components, including climate drivers such as humidity and temperature (Deyle et al., 2016; He et al., 2013; Mahmud et al., 2023; Shaman & Kohn, 2009; Yang et al., 2015; Yuan et al., 2021), human mobility (Charu et al., 2017; Massad et al., 2010; Merler & Ajelli, 2010), and school terms (Earn et al., 2012; Simon et al., 2009). The combined effects of these factors can give rise to complex and dynamic infection rates. Here we adopt a flexible time-varying periodic infection rate, *β*(*t*), given by the following expression:

(2) β(t)=Asin(Bt+C)+D.

The parameters *B* and *C* correspond to the angular frequency and phase shift of the sine function, while *A* and *D* represent its amplitude and vertical shift. To parameterize *β*(*t*) in terms of its maximum and minimum values *β*_max_ and *β*_min_, we can simply write $A=\frac{\beta_{\text{max}}-\beta_{\text{min}}}{2}$ and $D=\frac{\beta_{\text{max}}+\beta_{\text{min}}}{2}$. Fig. 2 B) i) illustrates an example of a periodic *β*(*t*), representing periodic changes in the infection rate. The sinusoidal structure contains four interpretable parameters, of which three (*β*_max_, *B*, and *C*) are estimated through our model calibration procedure (see section *Fitting the model to H1N1 data*). By allowing the angular frequency *B* to be estimated rather than fixed, we do not commit *β*(*t*) to a specific period such as a 52-week cycle, accommodating transmission dynamics that may not align with strictly annual seasonality. The flexibility of the initial phase shift *C* is consistent with the geographic variability of the selected locations, which span tropical, temperate, and Southern Hemisphere regions.

*Loss-of-immunity rates.* The influenza virus evolves through two main processes: antigenic drift and antigenic shift (Kim et al., 2018). While antigenic shift can lead to the abrupt emergence of novel subtypes – such as the 2009 H1N1 pandemic strain – our modeling approach focuses on antigenic drift, the gradual accumulation of mutations that help the virus evade host immune responses (Altman et al., 2018; Van de Sandt et al., 2012; Wertheim, 2010). We represent the effects of antigenic drift using either a linear or a jump immunity decay model. For the linear model, we assume that the antigenic distance between circulating strains and the strains to which the population has acquired immunity steadily increases over time (Smith et al., 2004), eroding cross-protection in a continuous fashion. Since the Influenza A/H1N1 virus has been shown to exhibit a slower antigenic drift (Bedford et al., 2015), and the 2009 pandemic H1N1 has likewise remained comparatively antigenically stable (Guarnaccia et al., 2013), we consider a gradual, linearly increasing effect of viral mutation on the population-level immunity period. For the jump model, we assume that antigenic evolution proceeds in punctuated steps, in which the emergence of a new antigenic cluster abruptly returns a portion of recovered individuals to the susceptible class — a mechanism invoked to explain recurrent influenza outbreaks through sudden replenishment of the susceptible pool (Bock Axelsen et al., 2014; Yaari et al., 2013). The jump mechanism is consistent with evidence that a single mutation at a key site, such as on the hemagglutinin (HA) protein, can render the virus antigenically distinct (Centers for Disease Control and Prevention, 2022). Mathematically, we consider the following functions:

(3) $$\gamma \left(t\right)=\left(\frac{\gamma_{\text{max}}-\gamma_{0}}{T}\right)t+\gamma_{0}$$

and

(4) $$\gamma \left(t\right)=\gamma_{0}+\left(\gamma_{\text{max}}-\gamma_{0}\right)U\left(t-\tau \right).$$

For the linear model (Eq. (3)), *γ*_0_ and *γ*_max_ denote the baseline and maximum rates of loss of immunity, respectively, with *T* the interval over which *γ* rises from *γ*_0_ to *γ*_max_. We also set *T* in weeks based on the time between the initial outbreak and the year of the large resurgence in each location. Throughout this paper, we fix *γ*_0_ = 3.2 × 10^−3^ week^−1^ (see supplementary material for details). In the jump model (Eq. (4)), *γ*_max_ again defines the post-jump maximum loss rate, and *τ* specifies the time of the discrete increase. The term *U*(*t* − *τ*) represents a step function assuming the values 0 if *t* < *τ* and 1 if *t* ≥ *τ*. In what follows, we refer to the *linear immunity decay model* whenever the SVIRS model (Eq. (1)) is equipped with the linear function given by Eq. (3). Similarly, the *jump immunity decay model* refers to the SVIRS model with *γ*(*t*) given by Eq. (4).

*Vaccination rates.* The frequent antigenic changes prompt re-evaluations of influenza vaccines, which are typically administered yearly before the flu season. For example, in Brazil, vaccines are offered to high-risk groups through annual campaigns between April and May, a period that generally precedes the typical flu seasons (Stols Cruzeta et al., 2013). We adopted a time-varying vaccination rate *v*(*t*) as a rectangular pulse that achieves the maximum level *v*_max_ during the vaccination period for a given location (see Table S2 in the supplementary material). The underlying assumption behind the rectangular pulse is that doses are delivered within a time-limited annual window before the season rather than continuously throughout the year, reflecting the brief vaccination campaigns that precede the flu season. During this window, *v*(*t*) holds a constant value *v*_max_, which we interpret as the vaccination rate over the campaign and estimate from data for each location (see *Fitting the model to H1N1 data* below). For simplicity, the detailed temporal profile of uptake within the campaign is not modeled. Fig. 2 B) iii) illustrates the format of *v*(*t*).

## 3 Numerical simulations

**Fig. 3:**
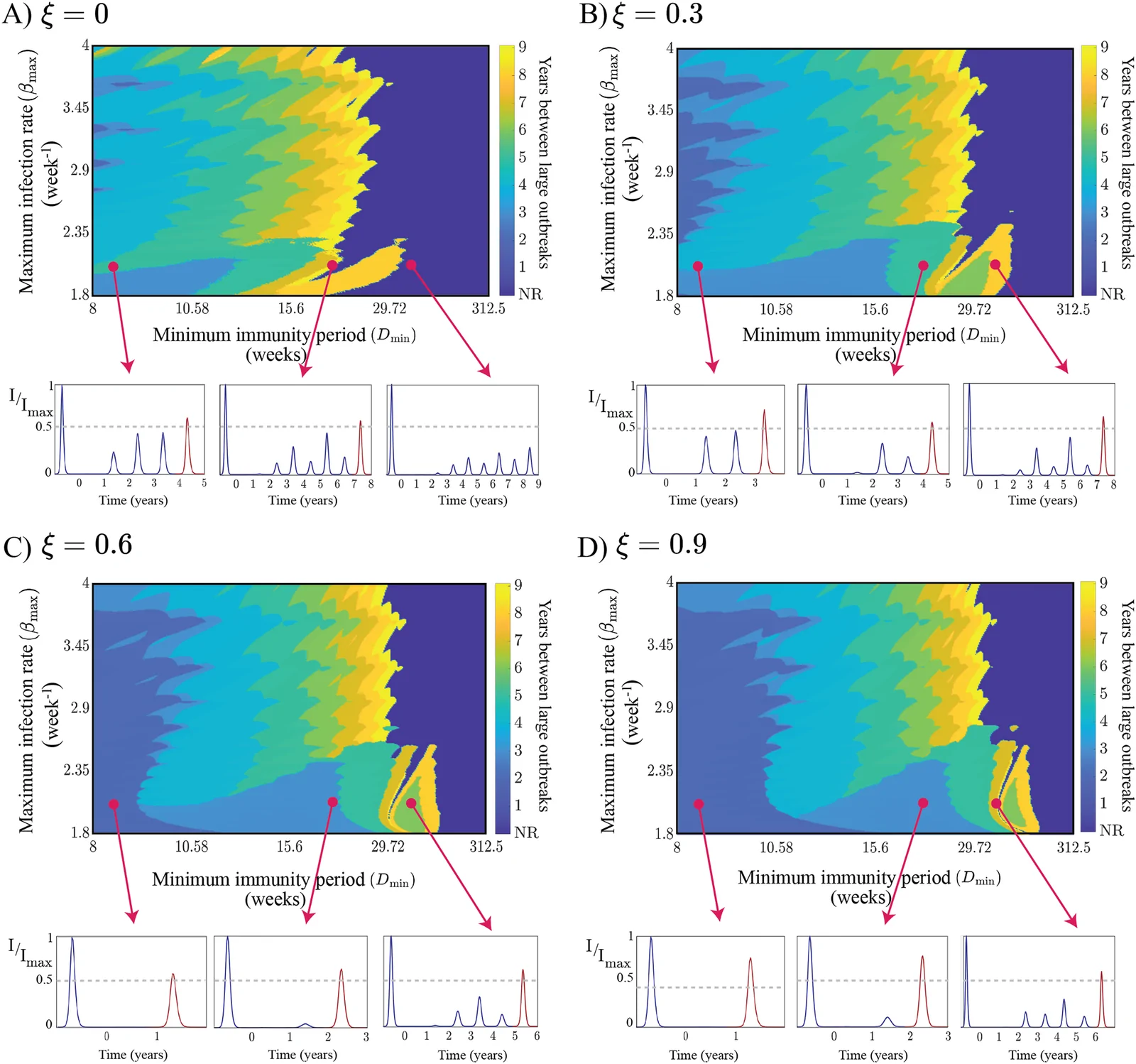
**Years between large outbreak peaks along with sample trajectories (linear immunity decay model).** Panels (A)–(D) show colormaps of the time interval (in years) between large outbreak peaks for increasing values of *ξ* (0, 0.3, 0.6, 0.9), corresponding to decreasing vaccine efficacy. Dark blue regions labeled “NR” indicate no large outbreaks within the past ten years. Beneath each colormap are sample trajectories of the normalized infectious population. Gray dashed lines indicate the threshold used to define large resurgent outbreaks (red colored). Parameter values were taken from Table 1. Initial population *N*_0_ = 42, 075, 716 and recruitment rate Λ = 137.9351 week^−1^ were taken from São Paulo, Brazil. Other fixed parameters include *β*_min_ = 1 week^−1^, *v*_max_ = 0.02 week^−1^, *γ*_0_ = 3.2 × 10^−3^ week^−1^, and *T* = 520 weeks (10 years). For the periodic infection rate, we set *B* = −0.1202 week^−1^ and *C* = −10.1360 to produce annual cycles.

We performed numerical simulations to explore parametric regimes that influence the timing of large resurgent outbreaks and to evaluate our model's ability to capture the overall characteristics of reported H1N1 case time series. Fig. 3 displays four sets of simulations conducted under different vaccine efficacy factors (*ξ*), considering the linear immunity decay model (Eq. (3)). Results for *ξ* = 0, 0.3, 0.6, and 0.9 are shown in panels A)–D). The colormaps depict the time interval (in years) between large outbreak peaks as a function of the minimum immunity period $D_{\min}=\frac{1}{\gamma_{\max}}$ and the maximum infection rate *β*_max_. For all colormaps, the time interval between large outbreaks tends to increase as the immunity period lengthens. Lower vaccine efficacy (i.e., larger *ξ* values) resulted in shorter time intervals between large outbreaks. That can be observed as colormaps become darker as *ξ* increases. We also display sample model trajectories for selected points in the colormaps, which exhibit decreasing time intervals between large outbreaks as *ξ* increases. The leftmost time series of each colormap shows the modeled weekly H1N1 cases for low *β*_max_ and *D*_min_. The resulting time interval between the first and large resurgent outbreaks decreases from 4 years to 1 year, as *ξ* changes from 0 to 0.6. Within each colormap, longer immunity periods tend to delay the occurrence of large resurgent outbreaks. For instance, in Fig. 3 A), three sample trajectories are highlighted. At the lowest *D*_min_ (approximately 8 weeks (Sun et al., 2010)), a large resurgent outbreak occurs 4 years after the initial outbreak. In contrast, at the highest *D*_min_ value (approximately 30 weeks), no large resurgent outbreak is observed within ten years, as indicated by the dark blue region marked (NR).

**Fig. 4:**
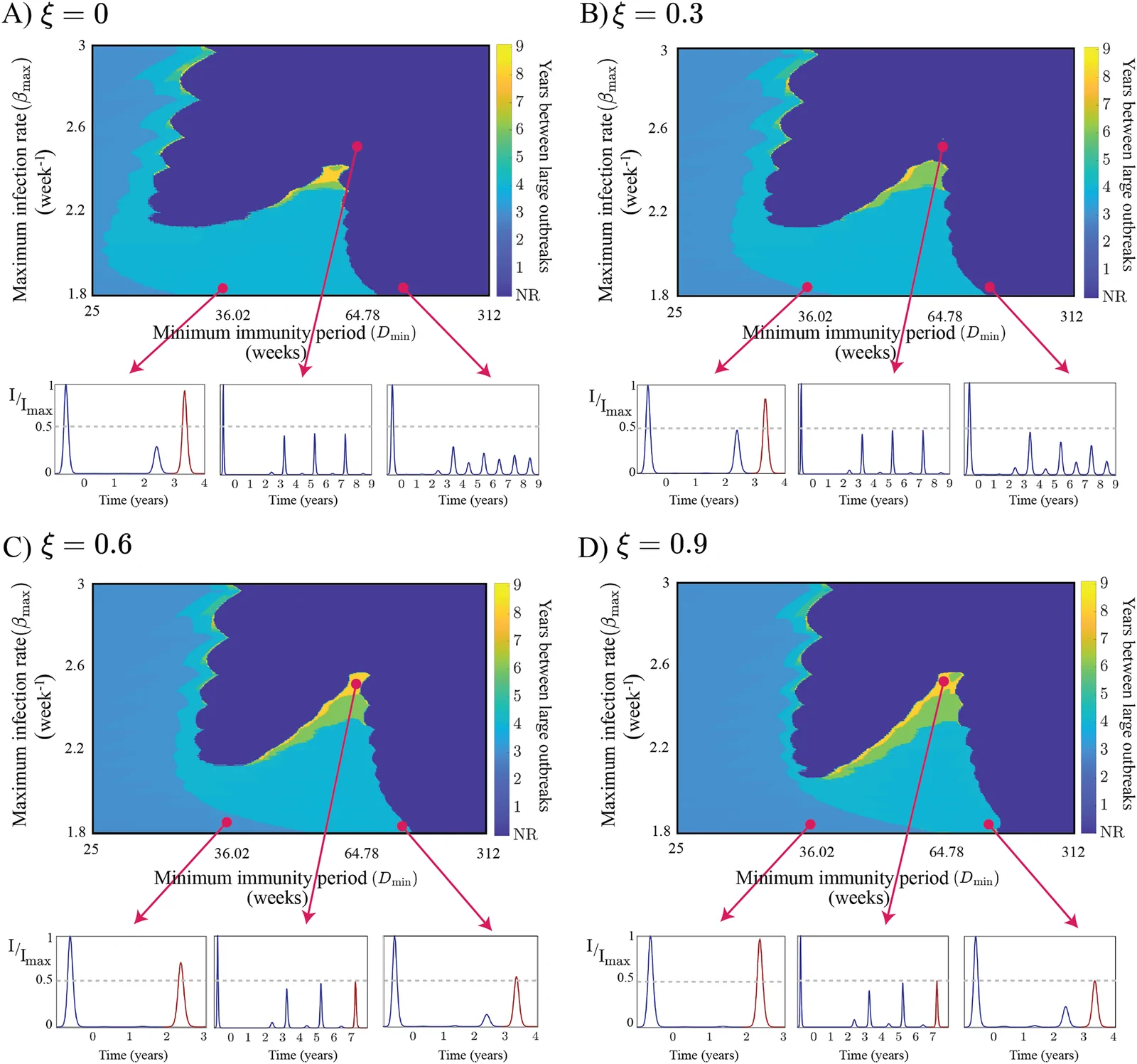
**Years between large outbreak peaks along with sample trajectories (jump immunity decay model).** Panels (A) - (D) exhibit four colormaps for increasing values of *ξ*, corresponding to decreasing vaccine efficacy levels. We considered four different *ξ* values (*ξ* = 0, 0.3, 0.6, and 0.9). Here, *ξ* = 0 represents a vaccine that confers perfect protection (panel A). Dark blue regions indicate no large resurgent outbreaks within ten years (labeled “NR”). Here, we set *τ* = 2.5 years and *v*_max_ = 0.01 week^−1^ while all other parameter values remain the same as in Fig. 3.

Fig. 4 shows numerical simulations of the jump immunity decay model (Eq. (4)). We set *τ* = 130 weeks (2.5 years), representing antigenic changes occurring 130 weeks after the initial outbreak, and *v*_max_ = 0.01 week^−1^ while all other parameters remain the same as in Fig. 3. The predominance of light blue colormap regions across panels A–D indicates immediate large resurgent outbreaks following the jump, while yellow regions highlight outbreaks delayed by several years. Sample trajectories below each colormap illustrate these varied dynamics; Notably, the middle time series in panels C and D exhibit resurgent outbreaks that slightly exceed the threshold (gray dashed line) approximately seven years after the initial wave.

## 4 Sensitivity of resurgent peak timing and intensity

**Fig. 5:**
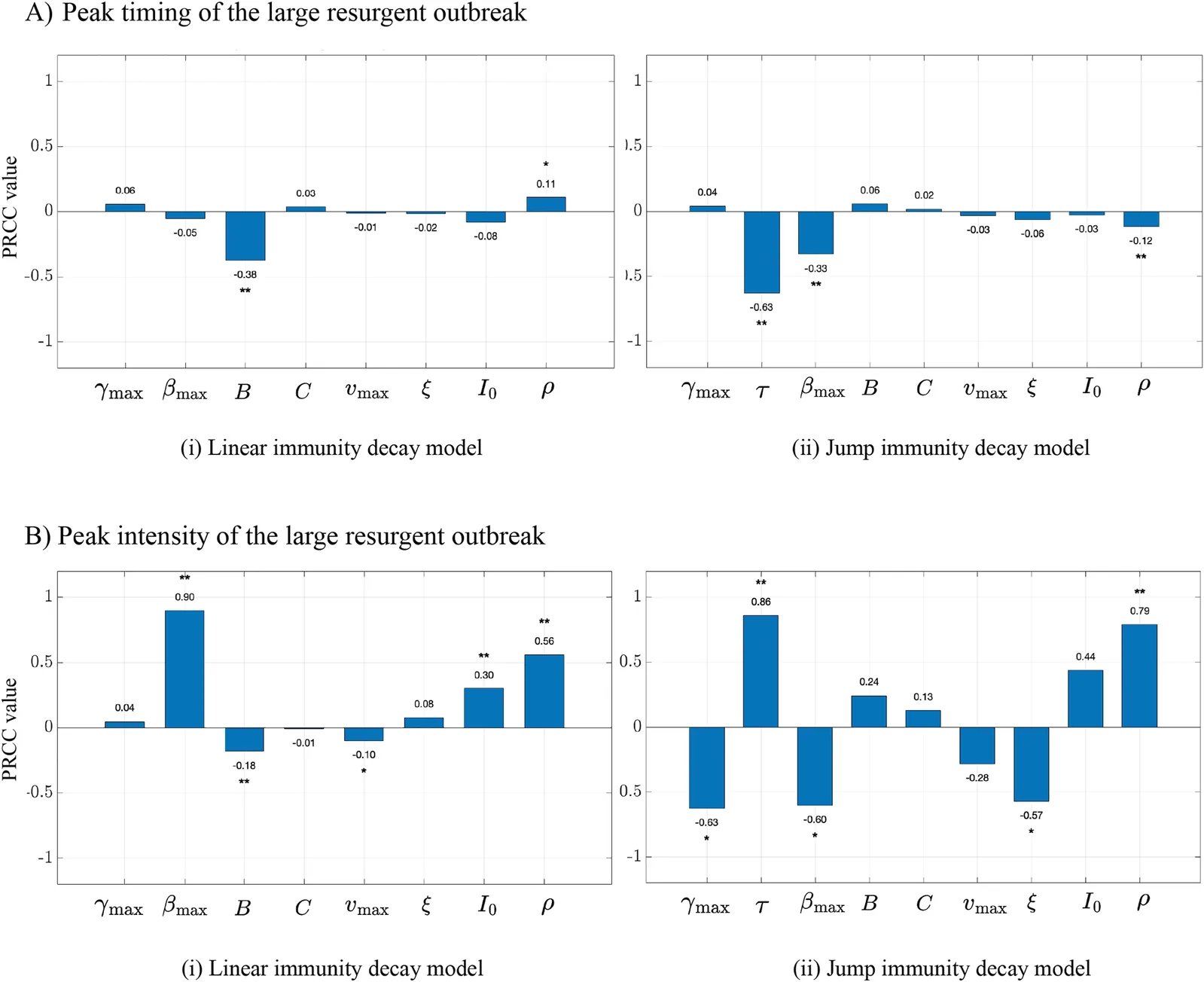
**PRCC sensitivity analysis of the model outputs to parameter values.** A) Peak timing and B) Peak intensity of the large resurgent outbreak for both linear and jump immunity decay models. Here, ∗ means *p* − *value* < 0.05 and ∗∗ means *p* − *value* < 0.01. Fixed parameter values taken from Table 1. Initial population *N*_0_ = 42, 075, 716 and recruitment rate Λ = 137.9351 week^−1^ taken from São Paulo, Brazil. We also fixed *β*_min_ = 1 week^−1^ < *r* to ensure natural epidemic decline in troughs of the infection rate.

To investigate the global sensitivity of the model outputs to the parameters, we used an LHS/PRCC approach (Marino et al., 2008), coupling Latin hypercube sampling (LHS) with partial rank correlation coefficients (PRCCs). Specifically, we explored the influence of each parameter on the peak and timing of the large resurgent outbreak. Using LHS, we generated a representative set of parameter combinations across the eight-dimensional parameter space for the linear model and the nine-dimensional space for the jump model. Parameter ranges were divided into N equally probable intervals using a uniform distribution. In this study, *N* = 500 parameter sets were generated. Using PRCC, we quantified the relationship between each input parameter and the model output obtained from the simulated reported infection curve, *ρI*(*t*). PRCC provides a value between −1 and 1 for each parameter, where positive values indicate that increasing the parameter tends to increase the output, negative values indicate that increasing the parameter tends to decrease the output, and values close to zero indicate weak correlation while controlling for the influence of all other parameters. The associated *p*-value measures whether this partial correlation is statistically significant. Therefore, parameters with large absolute PRCC values and small *p*-values are considered the most influential. Fig. 5 shows the results of our analysis.

For the linear immunity decay model, the angular frequency (*B*) exhibited a significant negative correlation with peak timing (PRCC = −0.38, *p* < 0.01; Fig. 5A(i)), indicating that higher oscillatory frequencies are associated with an earlier large resurgent peak. For the jump model, a similar negative effect was found for both the jump timing (*τ*) and the maximum transmission rate (*β*_max_) (Fig. 5A(ii)). Overall, the peak intensity exhibited a higher sensitivity to model parameters than the peak timing (Fig. 5B), suggesting that the magnitude of the outbreak is heavily driven by the combined influence of several parameters. For the linear model, the highest correlation with peak intensity was found for the maximum transmission rate (*β*_max_, PRCC = 0.90), reflecting the direct relationship between transmission potential and outbreak magnitude when immunity wanes gradually (Fig. 5B(i)). In contrast, for the jump model, the immunity jump timing (*τ*) exhibited the highest correlation (PRCC = 0.86), indicating that a longer interval before the immunity jump allows a larger pool of recovered individuals to accumulate; when the jump eventually occurs, this population becomes susceptible, fueling a more severe outbreak. Remarkably, within the jump model framework, the association between *β*_max_ and peak intensity was negative (PRCC = −0.60). This indicates that higher transmission rates may rapidly deplete the susceptible pool in the first outbreak, thus suppressing the magnitude of the subsequent resurgent outbreak in scenarios where immunity does not change gradually.

## 5 Fitting the model to H1N1 data

We evaluated our model's ability to fit H1N1 case data across the nine locations shown in Fig. 1 – São Paulo State in Brazil, Turkey, Iran, New Zealand, HHS Regions 2 and 6 in the US, Colombia, South Africa, and Croatia. These selected locations experienced a large resurgence of H1N1 outbreaks within a decade of the 2009 pandemic, a timeframe that captures the combined effects of waning immunity, cycles of higher transmission, and continued vaccination campaigns. For each location, we performed a least-squares optimization, fitting the simulated reported cases to the available case data.

### 5.1 Optimization procedure for model fitting

For the linear immunity decay model (Eqs. (1) and (3)), we estimated eight unknown parameters for each location. For simulations involving the jump immunity decay model (Eq. (4)), we estimated a total of nine parameters. See Table 2 for the list of the estimated parameters for each model. Other parameters were fixed at the values listed in Table 1, along with a fixed baseline loss-of-immunity rate *γ*_0_ = 3.2 × 10^−3^week^−1^ (see supplementary material). We also fixed *β*_min_ = 1week^−1^ so that during off-peak seasons we have *β*(*t*) < *r*, ensuring natural epidemic decline in troughs, while allowing *β*_max_ to drive seasonal surges.

**Table 2:** **List of the estimated parameters for each model.** Parameter estimation involved eight unknowns per location for the linear immunity decay model (Eqs. (1) and (3)) and nine for the jump immunity decay model (Eqs. (1) and (4)).

Parameter	Description	Immunity Decay Model
*β*_max_	Maximum infection rate	Linear and Jump
*γ*_max_	Maximum rates of loss of immunity	Linear and Jump
*v*_max_	Maximum vaccination rate	Linear and Jump
*τ*	Time where the jump occurs in the loss-of-immunity rate	Jump only
*B*	Angular frequency of *β*(*t*) function	Linear and Jump
*C*	Phase shift of *β*(*t*) function	Linear and Jump
*ξ*	Vaccine efficacy	Linear and Jump
*I*_0_	Initial infectious population	Linear and Jump
*ρ*	Reporting rate	Linear and Jump

To obtain the best-fit curves, we performed grid searches over the model parameters within predefined lower and upper bounds. For the linear immunity decay model, four evenly spaced values were assigned to each of the eight parameters, yielding 4^8^ = 65, 536 possible initial guesses. For each initial guess *θ*_*k*_, *k* = 1, 2, …, 4^8^, a least-squares optimal parameter value $\theta_{k}^{*}$ was obtained. Here, the objective function is the sum of squared residuals (SSR) given by $\text{SSR}\left(\theta \right)=\sum_{t=1}^{T}|y_{t}-{\hat{y}}_{t}\left(\theta \right){|}^{2}$ where *y*_*t*_ and ${\hat{y}}_{t}\left(\theta \right)$ denote the observed data and model-predicted values, respectively, and *T* is the total number of weekly observations for the given location. For simplicity, we considered ${\hat{y}}_{t}\left(\theta \right)=\rho I_{t}\left(\theta \right)$ under the assumption that modeled reported cases are a fraction of prevalence. Among all 4^8^ resulting optimal parameters $\theta_{k}^{*}$, we chose *θ*_opt_ as the one yielding the minimum SSR across iterations, i.e., $\theta_{\text{opt}}=\arg\min_{k}\left(\text{SSR}\left(\theta_{k}^{*}\right)\right)$. See Algorithm (1) in the supplementary material for further details. Optimization was performed using MATLAB's lsqcurvefit function. The system of ODEs was numerically integrated in MATLAB using the ode45 function, which employs a fourth-order Runge–Kutta method.

A similar procedure was applied to the jump immunity decay model, where three equally spaced initial guesses were used per parameter, resulting in 3^9^(19, 683) simulations. As this was a sparser search compared to the 4^8^ initial guesses used for the linear model, we conducted an additional 10,000 simulations using randomly sampled starting points to enhance the search for the global minimum.

To find the Uncertainty bounds for the best-fit curve for each location, we used a parametric bootstrapping approach as described in (Chowell, 2017). First, we computed the cumulative incidence curve *F*(*t*) defined by $F\left(t_{p}\right)=\sum_{k=1}^{p}z_{1}\left(t_{k}\right)$ where *z*_1_(*t*_*k*_) represents the number of cases in week *t*_*k*_. For all of our selected regions, the empirical variance of weekly cases is greater than the mean, indicating overdispersion. Hence, we added a negative binomial distribution to the best-fit curve to incorporate noise in the data. Precisely, new simulated incidence data were generated as *y*_*t*_ ∼ NB(*r*, *p*) where parameters *r* and *p* were derived from the empirical mean (*μ*_*t*_) and variance $\left(\sigma_{t}^{2}=\phi \mu_{t}\right)$ using $r=\frac{\mu_{t}^{2}}{\sigma_{t}^{2}-\mu_{t}}$ and $p=\frac{r}{r+\mu_{t}}$. For each location, a total of 100 realizations were generated for the model's best-fit using the negative binomial distribution. The model was subsequently refitted to every simulated dataset, with the best-fit set of parameters (*θ*_*opt*_) taken as the initial guess. For each of the 100 fits, we recorded the optimized parameter values and the associated residual sum of squares. From these replicates, 95% confidence intervals (CIs) for predicted incidence were computed as ${\text{CI}}_{95\%}\left(t\right)=\bar{y}\left(t\right)\pm 1.96\frac{s\left(t\right)}{\sqrt{n}}$ where $\bar{y}\left(t\right)$ and *s*(*t*) denote the mean and standard deviation across all bootstrapped curves at time *t*.

### 5.2 Best-fit curves and biological interpretation: linear immunity decay model

**Fig. 6:**
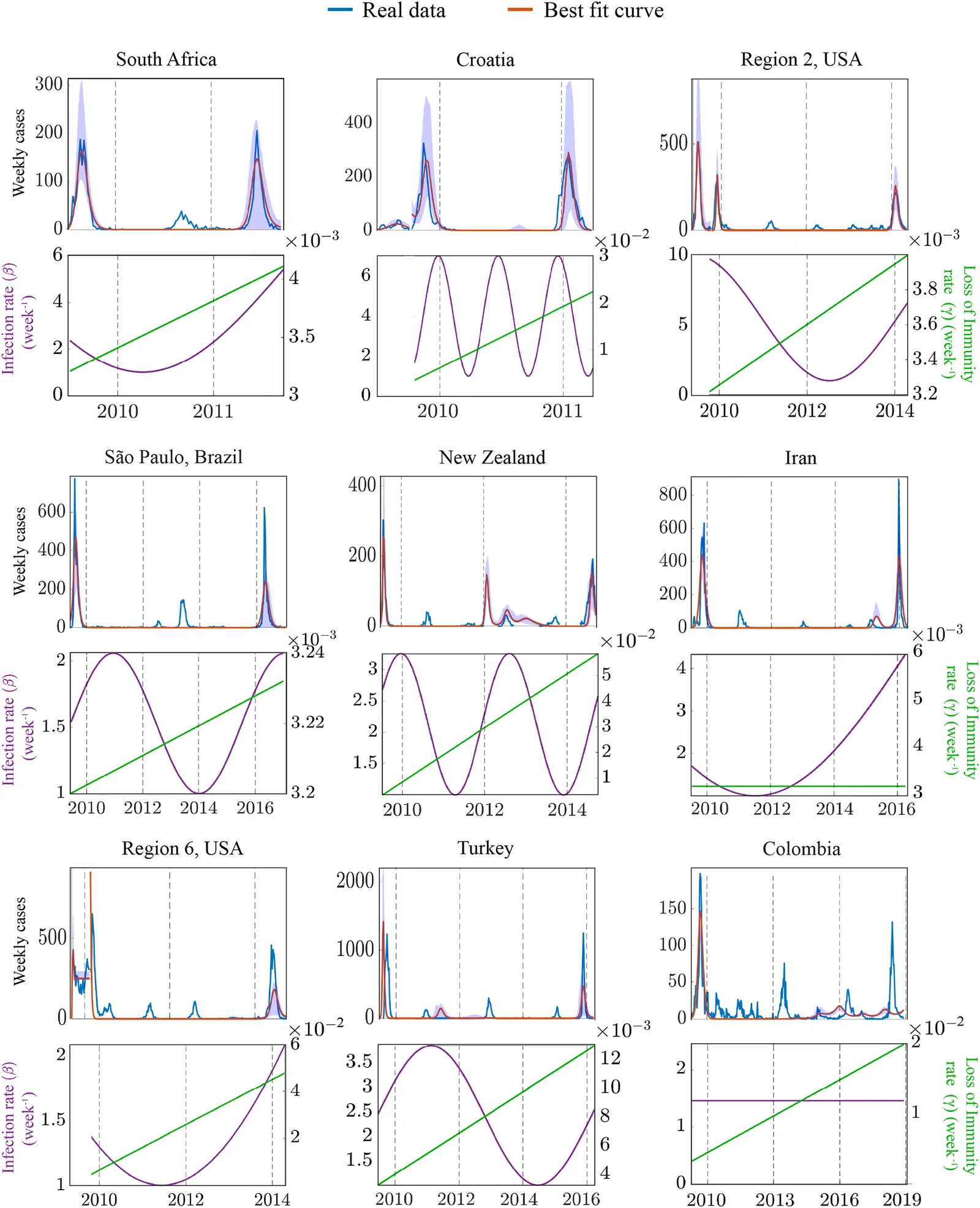
**Best-fit curves and optimal time-varying rates (linear immunity decay model).** The top panels show observed (blue) and modeled (red) weekly H1N1 cases for nine locations, with shaded uncertainty bounds. Bottom panels display the fitted infection rate *β*(*t*) (purple) and linearly increasing loss-of-immunity rate *γ*(*t*) (green).

The best-fit curves for the linear immunity decay model are presented in Fig. 6. Uncertainty bounds, shown in purple shades, were obtained as discussed above. Overall, the linear immunity decay model accurately captured the timing of most initial and large resurgent outbreaks. The closest fits occurred in South Africa, Croatia, and HHS Region 2 (USA). The model fits also showed reasonable agreement for São Paulo, New Zealand, and Iran, although sometimes over- or underestimating outbreak magnitudes. Remarkably, despite substantial gaps in the New Zealand data, the model recovered the large outbreaks and even inferred a surge around 2012 during the unreported interval. In contrast, mid-sized surges were less accurately captured in other locations. Model performance was slightly weaker in HHS Region 6 (USA), Turkey, and Colombia; notably, Colombia was the only location where the model failed to capture a clear resurgent wave, likely reflecting higher noise in the reported data. HHS Regions 2 and 6 in the United States experienced two pronounced H1N1 waves—in 2009 and again in 2010. Croatia followed a similar trajectory, with a modest preliminary wave occurring before its major 2009 outbreak. To account for these early peaks in 2009, we fitted the first outbreak to an *SIRS* model with constant rates of infection and loss of immunity. The resulting values of *S*, *I*, and *R*, together with *V* = 0, were then used as initial conditions for a second simulation using our SVIRS model, targeting the period between the 2009–2010 waves and the later large resurgence.

At the bottom of each best-fit panel in Fig. 6, the estimated rates of infection and loss of immunity illustrate the interplay between these two factors in shaping H1N1 dynamics up to the large resurgent outbreak. A flexible range of oscillations in the infection rate (dark blue) was observed, suggesting that complex transmission spikes arise from factors beyond seasonal influences. This implies that climate-driven oscillations alone may not fully explain the intricate temporal patterns of H1N1 outbreaks, particularly the resurgence events. For example, Croatia exhibited pronounced increases in infection rates during the winter flu season months (vertical dashed lines aligned with the beginning of each year), consistent with expected Northern Hemisphere seasonality. It also displayed a summer spike likely reflecting increased contact patterns due to tourism and summer activities. However, no outbreak was observed in the data nor in our best-fit solution, suggesting the susceptible pool was insufficient to sustain transmission despite elevated *β*(*t*). In contrast, South Africa did not show marked oscillations in *β*(*t*), but rather a gradual increase in transmission, possibly caused by the resumption of normal contact patterns following the pandemic year. An interesting decrease-followed-by-increase profile was found in HHS Regions 2 and 6 (USA), possibly reflecting the initial decrease due to residual behavioral caution, with a subsequent rise indicating the waning of protective measures.

For locations where the model fits were less accurate, biological interpretations should be made with caution. Nonetheless, reasonable fits for São Paulo and New Zealand in the Southern Hemisphere exhibited oscillations that are unlikely to be explained by the same mechanisms driving Northern Hemisphere dynamics. In São Paulo, peaks of high transmissibility were estimated in 2011 and 2016, the latter coinciding with the resurgent outbreak. New Zealand exhibited a sharp increase in *β*(*t*) during 2012–2013, allowing the model to capture one of the minor waves observed in the data and, interestingly, projecting a larger wave in 2012– a period for which no surveillance data is available.

Loss-of-immunity rates *γ*(*t*) are plotted in green on a separate y-axis.

In South Africa, *γ*(*t*) increased from the baseline value of 3.2 × 10^−3^ to approximately 4 × 10^−3^week^−1^, corresponding to a decrease in the immunity period from 312 weeks (6 years) to 250 weeks (4.8 years)—a modest reduction consistent with gradual antigenic drift rather than a punctuated immune escape event. A similar pattern was observed for HHS Region 2. In contrast, a more aggressive decline in immune protection was estimated for Croatia, New Zealand, and HHS Region 6. For example, in Croatia, *γ*(*t*) reached levels of 2 × 10^−2^week^−1^, indicating a decrease in the immunity period to approximately one year, possibly reflecting more rapid antigenic drift in the circulating strains in that region.

Notably, Iran's panel shows a virtually flat *γ*(*t*) – no appreciable increase in immunity loss –, yet the model still achieves a good fit. This suggests that our model can infer a possible absence of a significant antigenic drift over the considered period. All estimated parameters and confidence intervals can be found in the supplementary material (Table S4–S15).

### 5.3 Best-fit curves and biological interpretation: jump immunity decay model

**Fig. 7:**
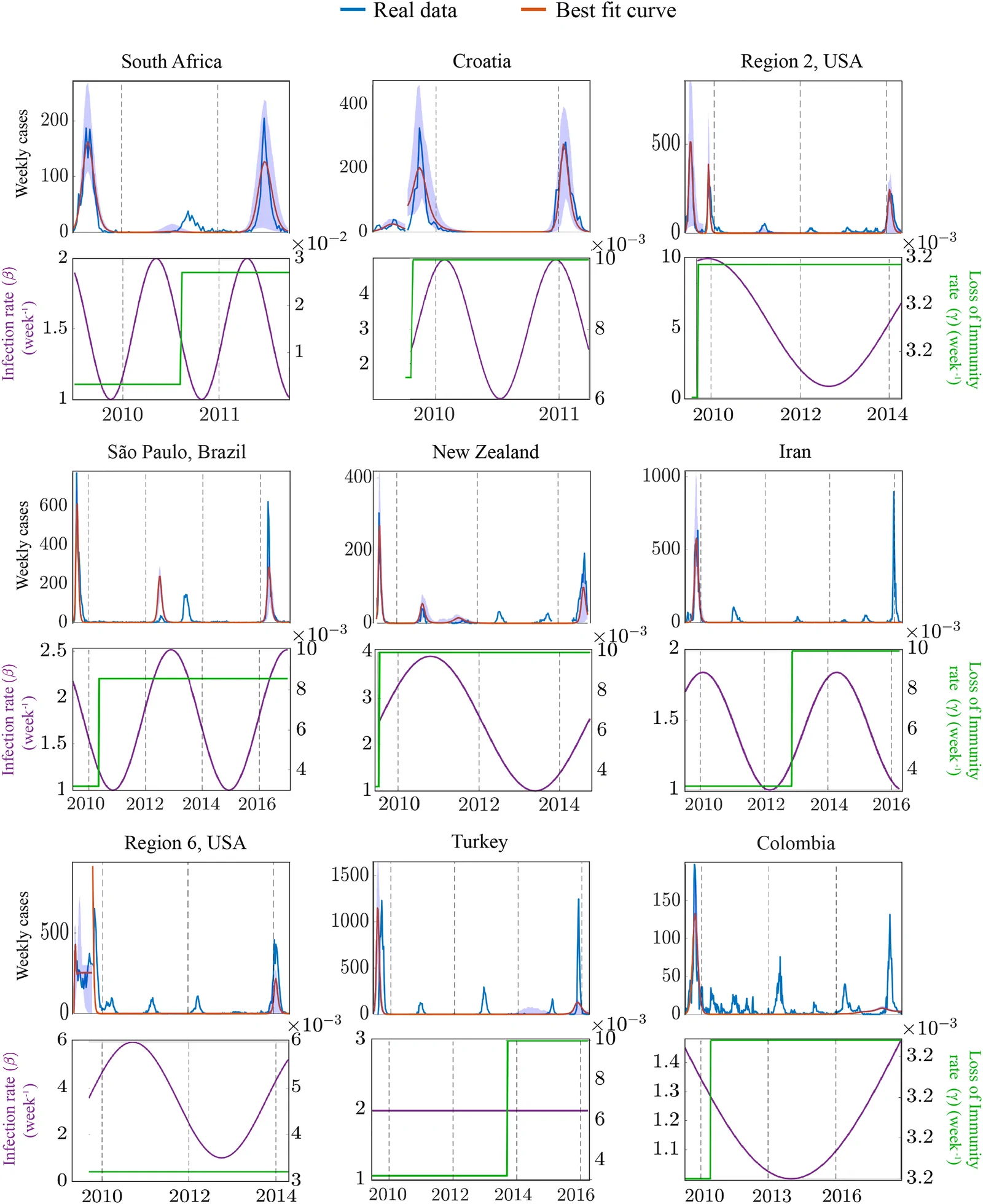
**Best-fit curves and time-varying rates (jump immunity decay model).** Top panels show observed (blue) and modeled (red) weekly H1N1 cases for nine locations, with shaded uncertainty bounds. Bottom panels display the fitted infection rate *β*(*t*) (purple) and jump loss-of-immunity rate *γ*(*t*) (green).

Fig. 7 presents a similar set of best-fit results for the jump immunity decay model. The model captured the overall timing and magnitude of large resurgent outbreaks in most locations. The bottom panels display the fitted rates of infection and loss of immunity. Interestingly, the jump immunity decay model enabled exploration of antigenic change timing. For instance, in South Africa, a jump was inferred between 2010 and 2011, reasonably close to the resurgent outbreak. In contrast, in São Paulo, the jump occurred closer to 2010, while the resurgent outbreak only took place in 2016. One possible interpretation is that antigenic changes preceding a large resurgent outbreak by several years may not immediately trigger resurgences if the susceptible pool has not yet been sufficiently rebuilt. However, the early jump timing may also reflect the model's structural constraint of a single jump, effectively approximating a sustained elevated loss-of-immunity rate over the full period. In some locations, such as Croatia and HHS Region 2 (USA), the best-fit solutions exhibited jumps at the beginning of the time series, suggesting that a constant immunity decay model might better describe influenza dynamics in these regions. Biologically, this could represent scenarios where immunity loss remained stable over time due to potentially mild antigenic changes.

Oscillation periods of *β*(*t*) varied by location, as in the linear immunity decay model. Best-fit solutions with higher-frequency *β*(*t*) oscillations were observed in South Africa, Croatia, and São Paulo, while lower-frequency oscillations were found in HHS Regions 2 and 6 (USA). As in the linear immunity decay model, higher-frequency oscillations may reflect regions where transmission is modulated by multiple factors within a single year, such as climate variability and distinct waves of social activity, whereas lower-frequency oscillations suggest more stable transmission dynamics or a lack of pronounced seasonality. South Africa exhibited good fits under a broader range of oscillatory patterns and *γ* values than those obtained with the linear immunity decay model, suggesting that seasonal winter activity combined with punctuated antigenic drift may also explain the observed resurgent outbreak. Similarly, Croatia displayed pronounced winter peaks consistent with the linear model; however, the best-fit *β*(*t*) did not include the summer peak inferred from the linear model. For São Paulo, the best-fit solution showed an increase in *β*(*t*) between 2012 and 2014, capturing one of the smaller outbreaks—albeit with a slight overestimation of its magnitude—that preceded the major resurgence in 2016.

## 6 Discussion

Large-scale resurgent H1N1 outbreaks have emerged worldwide, often reaching incidence levels comparable to those observed during the 2009 pandemic. To investigate these resurgences, we developed a mechanistic SVIRS model with assumptions grounded in biological and demographic drivers. Infection rates were chosen to fluctuate periodically to reflect the impact of environmental and social factors on transmission. We modeled loss of immunity dynamically using linear and jump functions to represent different effects of antigenic drift. Vaccination rates were represented by a time-varying pulse to reflect targeted seasonal campaigns. We investigated how different parameter combinations influence outbreak dynamics and assessed model fits to historical H1N1 data across nine locations.

Using numerical simulations (Fig. 3, Fig. 4), we identified parameter regimes that influence the timing of large resurgent outbreaks. Longer immunity durations increased the interval between large peaks, while reduced vaccine efficacy shortened these intervals. Our simulations indicate that periodic infection rates drive regular peaks in activity that escalate into large resurgent outbreaks only when the susceptible pool has been sufficiently replenished. Within this framework, severe resurgences arise from a conjunction of sustained transmission, loss of immunity, and insufficient vaccination. We fitted the linear and jump immunity decay models to historical H1N1 data from nine locations. Both models generally captured the timing of initial and resurgent outbreaks, though accuracy varied across locations. The closest fits occurred for South Africa, Croatia, and HHS Region 2 (USA), where the models effectively captured outbreak peaks. In contrast, fits for São Paulo, New Zealand, Iran, Turkey, and Colombia showed some discrepancies, particularly in capturing outbreak magnitudes or precisely aligning with observed peaks.

The fitted infection-rate oscillations varied considerably, suggesting that additional drivers beyond seasonal forcing may contribute to resurgent outbreaks. For example, results for the linear model in Croatia indicate a clear Northern Hemisphere winter peak and a summer rise in contacts that did not produce a resurgent outbreak, likely due to the lack of an adequate susceptible pool (Fig. 6). For South Africa, a monotonic increase can be interpreted as a return to normal contact patterns after the pandemic year. For both HHS regions in the United States, an initial dip followed by a rise in transmission may reflect complex dynamics in the absence of strong seasonal drivers. Estimated loss-of-immunity rates also spanned a range of profiles. Taking the linear model again as an example, we observed modest increases in *γ*(*t*) for South Africa and HHS Region 2, consistent with a mild effect of antigenic drift. In contrast, steeper slopes in the linear function were found for Croatia, New Zealand, and HHS Region 6, with Croatia reaching an immunity period of approximately one year. This diversity of transmission and immune regimes highlights the qualitatively different mechanisms driving large resurgent outbreaks that our model is capable of capturing.

In this study, two time-varying functions were proposed to model how waning immunity replenishes the susceptible pool. Previous work by Yaari et al. employed a more sophisticated version of the jump immunity decay model, incorporating multiple jumps corresponding to different influenza seasons to capture seasonal dynamics (Yaari et al., 2013). In contrast, our approach simplifies this idea by modeling immunity loss through a single jump or a linear increase. Biological evidence supports this simplification, as the H1N1pdm strain has remained antigenically stable in the years following the 2009 pandemic (Guarnaccia et al., 2013). To our knowledge, a time-dependent linear loss-of-immunity rate has not previously been used to model the effects of antigenic drift. Recent studies have adopted mechanistic models with multiple recovered stages to represent gradual waning immunity, each with a probability of reinfection (Andreu-Vilarroig et al., 2025; El Khalifi & Britton, 2023). In particular, El Khalifi and Britton (El Khalifi & Britton, 2023) proposed a linear decay function for the individual immunity level in terms of the average time since recovery, which is fundamentally different from the dynamic loss-of-immunity rate proposed in our model.

Our study has limitations. Parameter identifiability, which is typically assessed with profile likelihood analysis (Raue et al., 2009), Fisher Information Matrix (Miao et al., 2011; Roy Frieden, 2004), among other methods, was not formally evaluated in this study. Biological interpretations of the estimated parameters should thus be made with caution and not be regarded as definitive - our goal was to demonstrate that different interplays of time-varying transmission, immunity loss, and vaccination can explain a large resurgent H1N1 outbreak. While our model captured key features of large resurgent outbreaks, the modest fit in some locations suggests that further refinement is needed to better capture epidemic dynamics. For locations with many missing data points, such as New Zealand, fitting our models to extended data using imputation techniques remains an interesting exercise. However, handling unobserved intervals requires careful methodological considerations. For example, imputing highly nonstationary time series such as H1N1 case counts by relying on stationarity/seasonal regularity may not be well-justified. Still, future studies could validate the quality of the imputed data and explore the robustness of model fits against different imputation methods. Our current framework does not account for multi-strain dynamics, which are known to influence influenza patterns and may contribute to discrepancies in outbreak timing and magnitude (He et al., 2015; Minayev & Ferguson, 2009). Additional extensions could involve age-structured transmission, human mobility, and heterogeneous contact patterns (Ajelli et al., 2014; Balcan et al., 2009; Bansal et al., 2007). In this study, we adopted a parametric sine function for the infection rate, which constrains transmission dynamics to a fixed periodic structure and may not capture irregular shocks caused by behavioral changes, non-pharmaceutical interventions, or other factors. More flexible or nonparametric formulations, such as spline-based or data-driven approaches, could better capture irregular transmission patterns, though at the risk of introducing identifiability and over parametrization issues. Future studies could explore the trade-off between fitting accuracy and model complexity in depth.

## 7 Conclusions

We developed a mechanistic model to investigate large resurgent H1N1 outbreaks, integrating time-varying transmission, loss of immunity, and vaccination within a single framework. Using numerical simulations, we demonstrate that such large resurgences emerge from a conjunction of sustained transmission, gradual or punctuated loss of immunity, and reduced vaccination efficacy. By fitting the model to weekly surveillance data from nine geographically diverse locations, we found that our model overall captured the timing and principal features of large resurgent outbreaks. Fitted transmission rates exhibited a wide range of temporal profiles, suggesting that complex transmission spikes may arise from factors beyond seasonal forcing alone. Inferred loss-of-immunity rates also varied markedly across regions, pointing to distinct antigenic-drift effects.

Our modeling framework and insights, though developed in the context of H1N1 outbreaks, are broadly applicable to other influenza viruses with similar antigenic and seasonal characteristics. The interplay between transmission variability, waning immunity, and vaccination coverage is not unique to H1N1 and could help explain resurgence dynamics across different influenza subtypes. Future studies could extend our modeling approach to investigate influenza resurgences in the post COVID-19 pandemic era. The existence of an *immunity debt* caused by non-pharmaceutical interventions has been a topic of recent studies (L et al., 2023; James et al., 2025). Modeling efforts could, for example, combine the three time-varying functions proposed in our model with the effects of NPIs via data-informed reduction of contact rates. The resulting transmission rate would then include both periodic effects and discrete shocks driven by NPIs. Future research can also explore hybrid models that combine linear and jump components of the loss-of-immunity rate to capture biologically plausible regimes in which variants with few mutations are followed by those with many more, as observed in chronic SARS-CoV-2 infections (Zhou et al., 2023). Finally, incorporating genomic surveillance data to inform our loss-of-immunity rates is an exciting research direction. In particular, it remains important to determine whether an evolution-informed approach—as in Du et al. (Du et al., 2017)—would yield substantially different estimates for *γ*(*t*) if viral sequence data is available. In some scenarios, such as regions that experienced a single dominant antigenic change, we suspect that our simplified *γ*(*t*) functions may closely approximate more complex evolution-informed structures.

Our approach contributes to a broader understanding of how time-dependent biological factors shape the dynamics of respiratory infections. By exploring the effects of environmental variability, immune landscape evolution, and vaccination strategies within a single modeling framework, this work enhanced our understanding of large-scale influenza resurgence and may help inform future preparedness strategies.

## CRediT authorship contribution statement

**Chanaka Kottegoda:** Writing – review & editing, Writing – original draft, Formal analysis, Data curation. **Claudia T. Codeço:** Writing – review & editing, Conceptualization. **Claudio J. Struchiner:** Writing – review & editing, Conceptualization. **Lucas M. Stolerman:** Writing – review & editing, Writing – original draft, Supervision, Project administration, Conceptualization.

## Declaration of competing interest

The authors declare that they have no known competing financial interests or personal relationships that could have appeared to influence the work reported in this paper.
