# Supplementary material for: Resurgence of large-scale influenza A (H1N1) outbreaks: Modeling the interplay of transmission, loss of immunity, and vaccination
Source: Infect Dis Model. 2026 Aug 26;12(1):104–21. doi: 10.1016/j.idm.2026.08.001 (PMC13571244; doi:10.1016/j.idm.2026.08.001)
Supplement: Supplementary file 1 [file mmc1.pdf]

Supplementary Material

The supplementary material details the estimation of fixed (time-independent) model parameters, our model fitting algorithm, the parameters obtained from least-squares optimization, a summary of model performances, and a link to the data and code used in this study. Reference numbers cited throughout refer to the bibliography of the main manuscript.

Time-independent model parameters

Population data

To estimate the recruitment rate Λ into the susceptible class, we fitted a linear model to population data from 2009 onward. For each location, the model was initialized with the recorded 2009 population (N_0), and the slope was estimated using the method of least squares, minimizing the sum of squared errors between the model and the observed data up to the year of the large resurgence (see Fig. S1). Overall, population changes were gradual and well approximated by a linear trend across all locations. The estimated growth rates, expressed in week^{-1}, are summarized in Table S1.

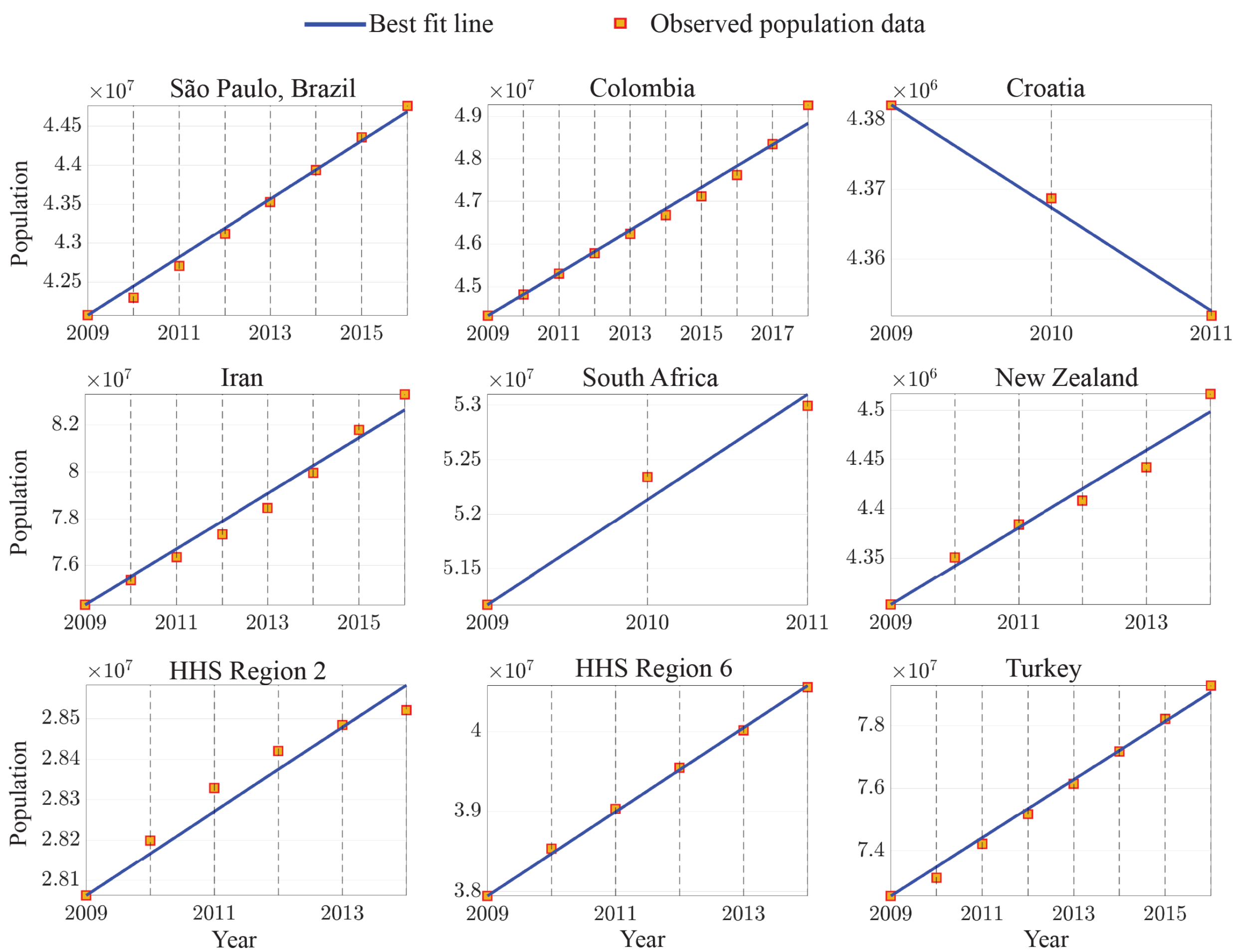

Figure S1: Population data for each location with best-fit linear curve

Location	Population in 2009 (N_0)	Growth rate Λ (week^{-1})
São Paulo, Brazil	$42,075,716$	137.9351
Colombia	$44,313,917$	186.0822
Croatia	$4,382,130$	-5.4751
Iran	$74,322,685$	439.9057
New Zealand	$4,302,600$	14.4896
HHS Region 2	$28,062,668$	38.4428
HHS Region 6	$37,944,626$	194.0172
South Africa	$51,170,779$	356.6660
Turkey	$72,561,312$	343.8197

Table S1: **Population in 2009 and estimated growth rates.**

Baseline loss-of-immunity rate (γ_0)

The constant parameter γ_0 represents the baseline rate at which individuals in the recovered class lose immunity. Its reciprocal, $\frac{1}{\gamma_0}$, corresponds to the baseline immunity duration, which we use to estimate the loss-of-immunity rate in 2009. Based on influenza studies reporting immunity durations ranging from 4 to 8 years [20], we assumed an average duration of 6 years. Converting to a weekly scale yields

$$\gamma_0 = \frac{1}{6 \times 52} = 3.2 \times 10^{-3} \text{ week}^{-1}.$$

Waning rate of vaccine-induced immunity (ω) and vaccination periods

The reciprocal $\frac{1}{\omega}$ represents the average duration of vaccine-induced immunity for an individual. In this study, we assumed 52 weeks based on [53], leading to

$$\omega = \frac{1}{52} = 0.019 \text{ week}^{-1}.$$

Vaccination periods for each location were estimated based on the months preceding winter and are summarized in Table S2.

**Location**	**Winter period**	**Estimated vaccination period**
Brazil	June - August	April - May
Colombia	December - February*	October - November
Croatia	December - February	October - November
New Zealand	June - August	April - May
Iran	December - February	October - November
Turkey	December - February	October - November
South Africa	June - August	April - May
HHS Region 2	December - February	October - November
HHS Region 6	December - February	October - November

Table S2: **Estimated vaccination periods.** Winter periods were defined according to the typical winter months in each region, accounting for their respective hemispheres and climate zones. In Colombia, where a true winter season does not occur due to its tropical climate, the rainy season was considered the equivalent of winter.

Recovery rate (r)

The reciprocal $\frac{1}{r}$ represents the average infectious period, typically ranging from 5 to 7 days [51]. We assumed an average duration of 6 days, which corresponds to $r = \frac{1}{0.85} = 1.17$ week^{-1}.

Model fitting algorithm

Algorithm (1) describes the process we use to obtain the best-fit parameter values for a given region with the linear immunity decay model. As for the jump model, we modified the algorithm (1) to include the time of the jump of the γ function (τ) as the fitted parameter with the lower bound 1 and the upper bound the time span (T) of the fitted region.

Algorithm 1 Grid Search Parameter Optimization for Model Fitting

Input: Population size N, time span T, observed data `cases`, initial population data `Pop_data` and linear model `model`.
Fixed Parameters:
Recovery rate $r = 1.17$; population growth rate Λ (see table S1) ; importation rate $\phi = 10^{-6}$; immunity waning rate $\omega = 1.9/100$; initial vaccination rate 10^{-8}; baseline loss-of-immunity rate $\gamma_0 = 3.2/1000$; vaccine effectiveness $\epsilon = 0.7$.
Fitted Parameters:
Maximum infection rate β_{max}; maximum rates of loss of immunity $\gamma_{\max}$; Angular frequency of $\beta(t)$ function B; Phase shift of $\beta(t)$ function C; Maximum vaccination rate $v_{\max}$; Vaccine efficacy ξ; Reporting rate ρ; Initial infectious population I_0.
Set parameter bounds:
Lower bounds $\mathbf{lb} = [\gamma_0, 1, 0, 0, 0.005, 0, 1, 0.0001]$.
Upper bounds $\mathbf{ub} = [0.125, 10, 2\pi, 2\pi, 0.08, 0.5, 1000, 0.05]$.
Define optimization settings:
Use `lsqcurvefit` with maximum 4000 evaluations, 50 iterations, Levenberg–Marquardt algorithm, and step tolerance 10^{-10}.
Generate parameter grids:
Set number of grid steps per parameter $n_{\text{step}} = 4$.
For each parameter p_i, construct grid $G_i = \text{linspace}(lb_i, ub_i, n_{\text{step}})$.
Initialize empty matrices $\mathbf{X}$ (parameter estimates) and $\mathbf{F}$ (objective values); set `loop_count` = 0.
for $i, j, k, l, o, p, q, s = 1, 2, \ldots, n_{\text{step}}$ **do**
Construct initial parameter guess:

$$\boldsymbol{\theta}_0 = [G_1(i), G_2(j), G_3(k), G_4(l), G_5(o), G_6(p), G_7(q), G_8(s)].$$

Perform curve fitting using `lsqcurvefit`:

$$(\boldsymbol{\theta}^*, f_{\text{val}}) = \texttt{lsqcurvefit}(\texttt{model}, \boldsymbol{\theta}_0, T, \texttt{cases}, \mathbf{lb}, \mathbf{ub}, \texttt{options}).$$

Store results: increment `loop_count`, and append $\boldsymbol{\theta}^*$ to $\mathbf{X}$, f_{val} to $\mathbf{F}$.
end for
Find optimal parameters:
Identify index r of minimum objective value:

$$[f_{\min}, r] = \min(\mathbf{F})$$

Identify best-fit parameter values:

$$\boldsymbol{\theta}_{\text{opt}} = \mathbf{X}(r, :).$$

Output: Optimal parameter vector $\boldsymbol{\theta}_{\text{opt}}$ and minimal objective value $f_{\min}$.

Table S3 displays the percentage of trajectories from the grid search optimization (Algorithm (1)) for which the optimized parameters θ^* were close to the global optimum θ_{opt}. Since the parameter ranges differed across variables, we defined the tolerance for each parameter as one-tenth of its

respective range:

$$\text{tol}_i = \frac{1}{10}(ub_i - lb_i),$$

where ub_i and lb_i denote the upper and lower bounds for the i-th parameter, respectively. A solution θ^* was considered close to θ_{opt} if $|\theta_i^* - \theta_{\text{opt},i}| < \text{tol}_i$ for all parameters i. The percentage reported represents the proportion of such solutions relative to the total number of optimization runs. The small percentages suggest that the global optimum occupies a narrow region in parameter space, with most optimization runs converging to local minima.

Location	Immunity Decay Model	Convergence to optimum (%)
São Paulo, Brazil	linear	0.0916%
	jump	0.0034%
Colombia	linear	0.0122%
	jump	0.0033%
Croatia	linear	0.0122%
	jump	0.6944%
Iran	linear	0.0153%
	jump	0.0034%
New Zealand	linear	0.0168%
	jump	0.75801%
South Africa	linear	0.009%
	jump	0.0067%
Turkey	linear	0.15%
	jump	0.0034%
HHS Region 2, USA	linear	0.0183%
	jump	0.0060%
HHS Region 6, USA	linear	0.0061%
	jump	0.0060%

Table S3: **Percentage of optimization runs converging to the global optimum.**

Estimated parameters from least-squares optimization and confidence intervals

Brazil

Parameter	Immunity Decay Model	Best-fit value	95% CI
$\gamma_{\max}$	linear	0.003232	$[0.0032, 0.003237]$
	jump	0.0086	$[0.0075, 0.0094]$
$\beta_{\max}$	linear	2.0598	$[2.0597, 2.0704]$
	jump	2.5160	$[2.5157, 2.5163]$
Angular frequency of $\beta(t)$	linear	0.01976	$[0.0146,\ 0.0198]$
	jump	0.0295	$[0.0291, 0.03]$
Phase of $\beta(t)$	linear	6.2822	$[6.2818,\ 6.2832]$
	jump	2.5244	$[2.5242, 2.5248]$
$v_{\max}$	linear	0.005	$[0.005,\ 0.005002]$
	jump	0.005	$[0.005, 0.0099]$
ξ	linear	0.5	$[0.4999995,\ 0.5]$
	jump	6.7×10^{-11}	$[0, 0.05 \times 10^{-4}]$
ρ	linear	2.5466×10^{-4}	$[1.360 \times 10^{-4},\ 3.273 \times 10^{-4}]$
	jump	1.3675×10^{-4}	$[1 \times 10^{-4},\ 1.6615 \times 10^{-4}]$
$I0$	linear	38.0612	$[7.6354,\ 57.5562]$
	jump	1	$[1,\ 1.7092]$
τ	jump	48.8403	$[48.8403, 48.8403]$

Table S4: **Parameter estimates for São Paulo, Brazil.** Best-fit values and 95% CIs obtained from parametric bootstrapping.

Colombia

Parameter	Immunity Decay Model	Best-fit value	95% CI
$\gamma_{\max}$	linear	0.02	$[0.0170, 0.0242]$
	jump	0.003217	$[0.0032, 0.003236]$
$\beta_{\max}$	linear	10	$[9.998, 10]$
	jump	1.9094	$[1.9094, 1.9095]$
Angular frequency of $\beta(t)$	linear	0	$[0, 1.148 \times 10^{-6}]$
	jump	6.2768	$[6.2766, 6.2769]$
Phase shift of $\beta(t)$	linear	4.255629	$[4.255623, 4.255632]$
	jump	0	$[0, 0.3059 \times 10^{-4}]$
$v_{\max}$	linear	0.0105	$[0.0100, 0.0116]$
	jump	0.01	$[0.01, 0.01]$
ξ	linear	0	$[0, 0.0721]$
	jump	0.499991	$[0.499985, 0.5]$
ρ	linear	1.5227×10^{-4}	$[1.294 \times 10^{-4},\ 1.544 \times 10^{-4}]$
	jump	5.5207×10^{-4}	$[1.2518 \times 10^{-4}, 2.0681 \times 10^{-4}]$
$I0$	linear	1.1011	$[1,\ 1.2029]$
	jump	1	$[1,\ 1]$
τ	jump	60.3514	$[60.3514, 60.3514]$

Table S5: **Parameter estimates for Colombia.** Best-fit values and 95% CIs obtained from parametric bootstrapping.

Croatia

Parameter	Best-fit value	95% CI
γ	0	$[0, 0.2740]$
β	1.6199	$[1.4792, 1.9323]$
$I0$	2.0768	$[1.0000, 3.9320]$
ρ	0.0001	$[0.00005,\ 0.00241]$

Table S6: **Parameter estimates for Croatia with SIR model.** Best-fit values and 95% CIs obtained from parametric bootstrapping. Parameter estimates for Croatia for the first outbreak. Best-fit values and 95% CIs obtained from parametric bootstrapping. The SIR model was fitted to data from 29-06-2009 through 05-10-2009 (15 weeks).

Parameter	Immunity Decay Model	Best-fit value	95% CI
$\gamma_{\max}$	linear	0.0257	$[0.0109, 0.0356]$
	jump	0.0100	$[0.0076, 0.0100]$
$\beta_{\max}$	linear	7.05	$[5.7620, 8.4028]$
	jump	4.9944	$[4.7634, 5]$
Angular frequency of $\beta(t)$	linear	0.2398	$[0.2229, 0.2503]$
	jump	0.1290	$[0.1177, 0.1439]$
Phase shift of $\beta(t)$	linear	5.149	$[4.8536, 5.3873]$
	jump	5.8164	$[5.5225, 6.1334]$
$v_{\max}$	linear	0.08	$[0.005, 0.08]$
	jump	0.0134	$[0.005, 0.0248]$
ξ	linear	0.499596	$[0.2752, 0.5]$
	jump	0.5	$[0.4432, 0.5]$
ρ	linear	8.0991×10^{-4}	$[5 \times 10^{-4},\ 13 \times 10^{-4}]$
	jump	9.3802×10^{-4}	$[6.3501 \times 10^{-4}, 14.835 \times 10^{-4}]$
τ	jump	1	$[1, 1.0208]$

Table S7: **Parameter estimates for Croatia.** Best-fit values and 95% CIs obtained from parametric bootstrapping.

New Zealand

Parameter	Immunity Decay Model	Best-fit value	95% CI
$\gamma_{\max}$	linear	0.0587	$[0.0460, 0.0871]$
	jump	0.009992	$[0.0085, 0.01]$
$\beta_{\max}$	linear	3.2591	$[2.7834, 3.7041]$
	jump	3.9148	$[3.4540, 4]$
Angular frequency of $\beta(t)$	linear	0.0459	$[0.0450, 0.0473]$
	jump	0.0233	$[0.0224, 0.0254]$
Phase shift of $\beta(t)$	linear	0.4632	$[0.2947, 0.8597]$
	jump	6.2832	$[6.1373, 6.2832]$
$v_{\max}$	linear	0.005043	$[0.005, 0.0390]$
	jump	0.0050006	$[0.005, 0.0321]$
ξ	linear	0.5	$[0, 0.5]$
	jump	0.5	$[8.8846 \times 10^{-8}, 0.5]$
ρ	linear	2.9337×10^{-4}	$[2 \times 10^{-4},\ 5 \times 10^{-4}]$
	jump	3.3975×10^{-4}	$[2.1309 \times 10^{-4}, 5.5847 \times 10^{-4}]$
$I0$	linear	7.3363	$[1,\ 15.4336]$
	jump	11.3587	$[5.5131,\ 24.5351]$
τ	jump	1	$[1, 1]$

Table S8: **Parameter estimates for New Zealand.** Best-fit values and 95% CIs obtained from parametric bootstrapping.

Iran

Parameter	Immunity Decay Model	Best-fit value	95% CI
$\gamma_{\max}$	linear	0.0032	$[0.0032, 0.003205]$
	jump	0.01	$[0.0032, 0.01]$
$\beta_{\max}$	linear	10	$[9.9989, 10]$
	jump	1.8452	$[1.8066, 1.9531]$
Angular frequency of $\beta(t)$	linear	0.0042011	$[0.0041894, 0.00042371]$
	jump	0.0287	$[0, 0.1317]$
Phase shift of $\beta(t)$	linear	4.1555	$[4.1554, 4.1556]$
	jump	0.7030	$[0.1349, 0.7544]$
$v_{\max}$	linear	0.078921	$[0.078920, 0.08]$
	jump	0.08	$[0.0105, 0.08]$
ξ	linear	0.498885344	$[0.498885343, 0.5]$
	jump	0.0611	$[0.0003, 0.2404]$
ρ	linear	1.9486×10^{-4}	$[1.6146 \times 10^{-4},\ 2.0376 \times 10^{-4}]$
	jump	1.0876×10^{-4}	$[1 \times 10^{-4},\ 1.7370 \times 10^{-4}]$
$I0$	linear	1.008723	$[1.008662,\ 1.008728]$
	jump	1	$[1,\ 1]$
τ	jump	176	$[176, 176]$

Table S9: **Parameter estimates for Iran.** Best-fit values and 95% CIs obtained from parametric bootstrapping.

Turkey

Parameter	Immunity Decay Model	Best-fit value	95% CI
$\gamma_{\max}$	linear	0.0129	$[0.0032, 0.0195]$
	jump	0.01	$[0.0032, 0.01]$
$\beta_{\max}$	linear	3.8400	$[3.8383, 3.8436]$
	jump	2.8783	$[2.8772, 2.9283]$
Angular frequency of $\beta(t)$	linear	0.0180	$[0.0165, 0.0185]$
	jump	0	$[0, 0.0006]$
Phase shift of $\beta(t)$	linear	6.282821	$[6.282770, 6.283185]$
	jump	0.0534	$[0.0522, 0.0535]$
$v_{\max}$	linear	0.005	$[0.005, 0.0153]$
	jump	0.08	$[0.005, 0.08]$
ξ	linear	0.5	$[0, 0.5]$
	jump	0.5	$[0, 0.5]$
ρ	linear	1.0556×10^{-4}	$[0.6695 \times 10^{-4},\ 1.8525 \times 10^{-4}]$
	jump	1.5917×10^{-4}	$[0.8372 \times 10^{-4},\ 2.2217 \times 10^{-4}]$
$I0$	linear	1	$[1,\ 2.8217]$
	jump	30.2896	$[3.9054,\ 70.5599]$
τ	jump	221.9301	$[221.9301, 221.9301]$

Table S10: **Parameter estimates for Turkey.** Best-fit values and 95% CIs obtained from parametric bootstrapping.

South Africa

Parameter	Immunity Decay Model	Best-fit value	95% CI
$\gamma_{\max}$	linear	0.0041	$[0.0032, 0.0189]$
	jump	0.027	$[0.0188, 0.0709]$
$\beta_{\max}$	linear	10	$[9.9991, 10]$
	jump	2	$[1.9199, 2]$
Angular frequency of $\beta(t)$	linear	0.0203	$[0.0193, 0.0213]$
	jump	0.1280	$[0.1237, 0.1313]$
Phase shift of $\beta(t)$	linear	3.8920	$[3.8518, 3.9115]$
	jump	2.0913	$[1.8203, 2.1139]$
$v_{\max}$	linear	0.0562	$[0.0453, 0.0757]$
	jump	0.0122	$[0.005, 0.0598]$
ξ	linear	0.0002	$[0, 0.0019]$
	jump	0.4995	$[0.4938, 0.5]$
ρ	linear	1.0240×10^{-4}	$[1 \times 10^{-4},\ 1.5390 \times 10^{-4}]$
	jump	1×10^{-4}	$[1 \times 10^{-4},\ 1.6379 \times 10^{-4}]$
$I0$	linear	1.5213	$[1.5149,\ 1.5260]$
	jump	6.2340	$[6.2307,\ 6.3432]$
τ	jump	59	$[59, 59]$

Table S11: **Parameter estimates for South Africa.** Best-fit values and 95% CIs obtained from parametric bootstrapping.

HHS Region 2

Parameter	Best-fit value	95% CI
γ	0	$[0, 0.0940]$
β	2.0166	$[1.8021, 2.7108]$
$I0$	10.8779	$[1.0000, 22.7164]$
ρ	0.0002	$[0.00015,\ 0.00031]$

Table S12: **Parameter estimates for HHS 2 (United States) for first outbreak with SIR model.** Best-fit values and 95% CIs obtained from parametric bootstrapping. The SIR model was fitted to data from epidemiological week 16, 2009, through epidemiological week 38, 2009 (23 weeks).

Parameter	Immunity Decay Model	Best-fit value	95% CI
$\gamma_{\max}$	linear	0.004	$[0.0032, 0.0055]$
	jump	0.0032	$[0.0032, 0.0034]$
$\beta_{\max}$	linear	9.9906	$[9.9769, 10]$
	jump	12.1608	$[11.8924, 12.3074]$
Angular frequency of $\beta(t)$	linear	0.0196	$[0.0120, 0.0199]$
	jump	0.0222	$[0.0215, 0.0240]$
Phase shift of $\beta(t)$	linear	1.8298	$[1.8297, 2.0105]$
	jump	1.2788	$[1.0519, 1.6021]$
$v_{\max}$	linear	0.0541	$[0.005, 0.0701]$
	jump	0.08	$[0.0549, 0.08]$
ξ	linear	0.5	$[0.0588, 0.5]$
	jump	0.4977	$[0.1149, 0.5]$
ρ	linear	1.8985×10^{-4}	$[1.1631 \times 10^{-4},\ 2.7578 \times 10^{-4}]$
	jump	1.7207×10^{-4}	$[1.1865 \times 10^{-4}, 2.8047 \times 10^{-4}]$
τ	jump	1	$[1, 1.0003]$

Table S13: **Parameter estimates for HHS 2 (United States)** Best-fit values and 95% CIs obtained from parametric bootstrapping.

HHS Region 6

Parameter	Best-fit value	95% CI
γ	0.4633	$[0.2755, 0.9383]$
β	7.7481	$[3.2031, 8.9560]$
$I0$	1	$[1, 42.6134]$
ρ	0.000027	$[0.0000274,\ 0.000044]$

Table S14: **Parameter estimates for HHS 6 (United States) for first outbreak with SIR model. Best-fit values and 95% CIs obtained from parametric bootstrapping.** The SIR model was fitted to data from epidemiological week 16, 2009, through epidemiological week 38, 2009 (23 weeks).

Parameter	Immunity Decay Model	Best-fit value	95% CI
$\gamma_{\max}$	linear	0.0480	$[0.0479, 0.0846]$
	jump	0.0032	$[0.0032, 0.0032]$
$\beta_{\max}$	linear	6.9894	$[1, 10]$
	jump	5.9994	$[5.9763, 6.0000]$
Angular frequency of $\beta(t)$	linear	0.0059	$[0, 0.006]$
	jump	0.0294	$[0.0293, 0.0295]$
Phase shift of $\beta(t)$	linear	4.1677	$[4.1674, 4.1682]$
	jump	0	$[0, 0.3169]$
$v_{\max}$	linear	0.0562	$[0.005, 0.08]$
	jump	0.0163	$[0.005, 0.08]$
ξ	linear	0.3330	$[0, 0.5]$
	jump	1×10^{-4}	$[0, 0.5]$
ρ	linear	1×10^{-4}	$[1 \times 10^{-4},\ 1.2675 \times 10^{-4}]$
	jump	1×10^{-4}	$[1 \times 10^{-4},\ 1.2475 \times 10^{-4}]$
τ	jump	239.0694	$[239.0694, 239.0694]$

Table S15: **Parameter estimates for HHS 6 (United States).** Best-fit values and 95% CIs obtained from parametric bootstrapping.

Model Performance

Model performance was evaluated using three different error metrics: Root Mean Square Error (RMSE), Mean Absolute Error (MAE), and symmetric mean absolute percentage error (SMAPE). The formulas for the corresponding metrics are given by

$$\text{RMSE} = \sqrt{\frac{1}{T}\sum_{t=1}^{T}(\hat{y}_t(\theta_{opt}) - y_t)^2}, \quad \text{MAE} = \frac{1}{T}\sum_{t=1}^{T}|\hat{y}_t(\theta_{opt}) - y_t|$$

and

$$\text{SMAPE} = \frac{2}{T}\sum_{t=1}^{T}\frac{|\hat{y}_t(\theta_{opt}) - y_t|}{|\hat{y}_t(\theta_{opt})| + |y_t|} \cdot 100\%,$$

where $\hat{y}_t(\theta_{opt})$ and y_t represent the model output and actual observed cases at time t, respectively. Here, T denotes the number of weeks. Error values for each location for both models are presented in the Table S16, with the lowest values highlighted in yellow. The linear immunity decay model outperformed the jump immunity decay model on all metrics for São Paulo and HHS Region 2 (USA), and at least two of the three metrics in all other locations, except Turkey, where the jump immunity decay model provided the best performance in terms of RMSE and MAE, although neither model captured the large outbreaks there particularly well. Overall, RMSE and MAE values were relatively low across most locations, indicating good fitting accuracy. Turkey was a notable exception, with much larger errors for both models, suggesting greater difficulty in capturing outbreak dynamics. Even if for most locations the preferred model differed depending on the metric, the differences in error values were small, indicating that both models provided comparable fits.

Location	Immunity Decay Model	RMSE	MAE	SMAPE
São Paulo, Brazil	linear	43.5778	13.8190	154.0165%
	jump	47.2714	16.7395	160.9063%
Colombia	linear	17.5558	9.7925	139.9145%
	jump	17.6997	9.1485	158.9455%
Croatia	linear	22.3348	11.1401	155.07175%
	jump	26.3099	12.9522	124.0109%
Iran	linear	49.7471	18.77895	180.6155%
	jump	67.4516	15.5259	188.0570%
New Zealand	linear	17.6085	10.0061	157.5454%
	jump	20.0159	10.6950	147.7273%
South Africa	linear	14.3537	8.3187	146.0223%
	jump	15.9148	9.4717	142.2827%
Turkey	linear	196.9188	63.2264	184.2504%
	jump	193.9137	58.9071	188.0306%
HHS Region 2, USA	linear	21.3360	10.9899	166.1053%
	jump	22.0727	11.9082	169.7987%
HHS Region 6, USA	linear	77.6263	29.6823	166.3314%
	jump	76.2881	30.0496	170.6639%

Table S16: **Model performance comparison (RMSE, MAE, and SMAPE).** Root Mean Square Error (RMSE), Mean Absolute Error (MAE), and Symmetric Mean Absolute Percentage Error (SMAPE) are reported for the linear and jump immunity decay models across all locations. The lowest error for each location is highlighted in yellow.

Data and Codes

All of the data and code are available at the following link.
